# DeepFLR facilitates false localization rate control in phosphoproteomics

**DOI:** 10.1038/s41467-023-38035-1

**Published:** 2023-04-20

**Authors:** Yu Zong, Yuxin Wang, Yi Yang, Dan Zhao, Xiaoqing Wang, Chengpin Shen, Liang Qiao

**Affiliations:** 1 Department of Chemistry, and Shanghai Stomatological Hospital, Fudan University, Shanghai, China; 2 Department of Computer Science, and Institute of Modern Languages and Linguistics, Fudan University, Shanghai, China; 3 Shanghai Omicsolution Co., Ltd, Shanghai, China

**Keywords:** Proteomic analysis, Data mining, Proteome informatics, Proteomics

## Abstract

Protein phosphorylation is a post-translational modification crucial for many cellular processes and protein functions. Accurate identification and quantification of protein phosphosites at the proteome-wide level are challenging, not least because efficient tools for protein phosphosite false localization rate (FLR) control are lacking. Here, we propose DeepFLR, a deep learning-based framework for controlling the FLR in phosphoproteomics. DeepFLR includes a phosphopeptide tandem mass spectrum (MS/MS) prediction module based on deep learning and an FLR assessment module based on a target-decoy approach. DeepFLR improves the accuracy of phosphopeptide MS/MS prediction compared to existing tools. Furthermore, DeepFLR estimates FLR accurately for both synthetic and biological datasets, and localizes more phosphosites than probability-based methods. DeepFLR is compatible with data from different organisms, instruments types, and both data-dependent and data-independent acquisition approaches, thus enabling FLR estimation for a broad range of phosphoproteomics experiments.

## Abstract (web summary)

Protein phosphorylation is a critical modification in many cellular processes. Here, the authors present DeepFLR, a deep learning-based framework to accurately predict phosphopeptide tandem mass spectra and effectively control false localization rates in phosphoproteomics.

## Introduction

Protein phosphorylation is widely implicated in cell signal pathways and regulates many cellular processes. The dysregulation of protein phosphorylation is commonly considered a hallmark of cancer and autoimmune diseases^1–3^. Phosphorylation can happen on different amino acid residues, typically serine (S), threonine (T), and tyrosine (Y). This common post-translational modification (PTM) is regulated by enzymes, like kinase and phosphatase, in a dynamic way. It is highly important to characterize the dynamic protein phosphorylation of a whole organism in an accurate and high throughput manner at the proteome-wide level. During the past years, phosphoproteomics has progressed rapidly, thanks to the technological advances in phosphopeptide enrichment, high-throughput mass spectrometry (MS), and computational proteomics^4^. In addition to the peptide sequence analysis in typical proteomics, it is crucial to determine phosphosite in phosphoproteomics, which, however, is still a challenge due to the coexistence of multiple candidate phosphorylation sites in one peptide, the lability of phosphate group during peptide fragmentation, and the limited quality of tandem mass spectra when analyzing highly complex samples^5,6^.

Despite the hurdles, a variety of phosphoproteome data analysis workflows have been developed. The essential idea is to match experimental tandem mass spectra to the theoretical fragment ions m/z. Then, phosphorylation site localization is performed based on the observation of site-determining fragment ions, and the probability of candidate phosphosites can be calculated by tools, such as AScore^7^, PTM score (MaxQuant/Andromeda)^8,9^, phosphoRS^10^, pSite^11^, etc. Otherwise, difference scores, including Mascot delta score (MD score)^12^, SLIP score^13^, and PepArML^14^, are calculated based on the identification scores given by search engines as an indication of the level of ambiguity in the site localization. Phosphosite localization based on the observation of site-determining fragment ions is usually hindered by the incompleteness of tandem mass spectra where no site-determining fragment ions are observed.

An alternative strategy is to match experimental spectra to the mass spectra in a library to select the best match result for phosphopeptide identification, which is not limited by the observation of site-determining fragment ions. However, the main challenge of the protocol is the spectral library building for phosphopeptides. One strategy of phosphopeptides spectral library construction is to generate semi-empirical tandem mass spectra of phosphopeptides from those of nonphosphorylated peptides by setting a mass shift of fragment peaks and considering phosphoric acid neutral loss (NL) from phosphorylated serine/threonine^15,16^. Another strategy attracting increasing interest is tandem mass spectra (MS/MS) prediction by machine learning. To date, there have already been a number of tools for the prediction of peptide MS/MS, including the representative conventional machine learning-based method MS^2^PIP^17^, and many deep learning-based methods, such as pDeep^18^, Prosit^19^, DeepMass:Prism^20^, DeepDIA^21^, etc. Nevertheless, there are only a few tools available for the prediction of phosphopeptides MS/MS. pDeep2 reports deep learning-based MS/MS prediction for peptides with PTMs including phosphorylation, which uses transfer learning to refine deep learning models for MS/MS prediction of phosphopeptides based on pre-trained models for unmodified peptides^22^. In 2021, Lou et al. developed DeepPhospho for accurate prediction of MS/MS of phosphopeptides, which adopts a hybrid network design including a bidirectional long short-term memory (Bi-LSTM) network for encoding peptides, a Transformer network for refining the peptide representation, and a regressor network for predicting fragment ion intensities^23^.

A common limitation of the current methods for phosphopeptide identification is the lack of an accurate estimation of false localization rate (FLR). Studies have shown that current tools follow disparate score distributions, leading to different FLRs for the same score by different tools^24,25^. Even for the same tool, an identical score can give rise to diverse FLRs for various datasets^24,25^. Thus, a recommended empirical score threshold by database searching or spectral matching does not universally applicable to real biological datasets for FLR control. So far, the most popular method to estimate FLR is to use synthetic phosphopeptides, which, however, is time-consuming and at a high cost. There have been tools introduced for phosphopeptides FLR control. LuciPHOr^5^ generates decoys by adding phosphate groups to all the non-candidate amino acid residues, i.e. any amino acids instead of S/T/Y, in a target peptide sequence. SLIP score^13^ published a decoy generation method of adding phosphorylated proline or glutamic acid during database searching to estimate FLR for a specific mouse dataset. To date, there are still not any universally acknowledged FLR control methods in phosphoproteomics. New methods are highly demanded that can accurately control FLR, accommodating the diversity of datasets, while having little impairment on detection sensitivity.

Herein, we present DeepFLR, a framework combining the deep learning-based phosphopeptide MS/MS prediction and a target-decoy approach for FLR control in phosphoproteomics. The deep-learning model for phosphopeptide MS/MS prediction is based on bidirectional encoder representations from Transformers (BERT)^26^ trained using >467,000 MS/MS of >184,000 phosphopeptides and >165,000 non-phosphopeptides. Compared to the existing phosphopeptide MS/MS spectra prediction tools, such as pDeep2^22^ and DeepPhospho^23^, our method can provide higher prediction accuracy as demonstrated using different datasets from *Homo sapiens*, *Mus musculus*, *Arabidopsis thaliana*, *Saccharomyces cerevisiae,* and *Escherichia coli*. A target-decoy strategy is developed for FLR control, where the decoys are generated by randomly exchanging the phosphorylated amino acid residue with another amino acid residue in the sequence, and the deep learning model is used to predict MS/MS for both target and decoy sequences. Identification of phosphopeptides is performed by matching the experimental spectra to the predicted spectra of both targets and decoys, and FLR estimation is performed based on the observation of decoys in the identification results. We benchmarked the DeepFLR on synthetic phosphopeptides datasets and biological datasets acquired by different types of instruments (orbitrap and Q-TOF). The results showed that DeepFLR can accurately estimate FLR and can lead to the identification of additional phosphosites. DeepFLR can perform analysis in combination with various database searching tools (e.g. MaxQuant, PEAKS, and SpectroMine), identify both monophosphopeptides and multiphosphopeptides, and assist in phosphoproteomics data analysis by both data-dependent acquisition (DDA) and data-independent acquisition (DIA) approaches.

## Results

### Deep learning model construction and performance evaluation for phosphopeptide MS/MS prediction

**Fig. 1:**
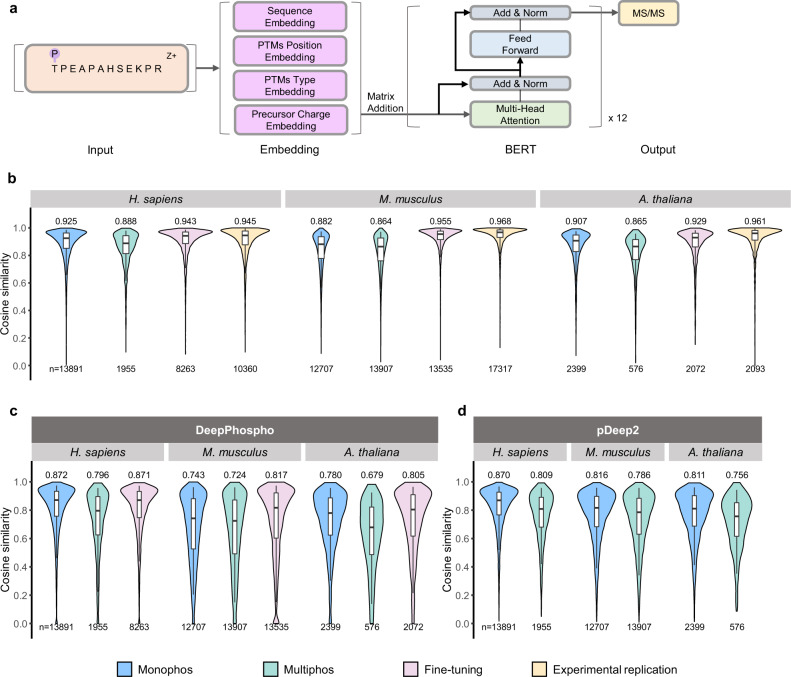
Model architecture and performance comparison. **a** The DeepFLR model architecture: input, the embedding layer, BERT encoder, and the output layer. The distribution of cosine similarity computed between the predicted and experimental spectra for **b** DeepFLR, **c** DeepPhospho, and **d** pDeep2. The cosine similarity of repeatedly collected mass spectra in the same dataset of the same phosphopeptides was also photted to demonstrate the performance of experimental variation. The medians are indicated. The boxes and whiskers show the quantiles and 95% percentiles, respectively. The numbers of spectra used are indicated below each graph. Missing values from DeepPhospho were inputted as 0. Source data are provided as a Source Data file.

The deep learning model for phosphopeptide MS/MS prediction consists of four components: input, the embedding layer, the BERT encoder and the output layer (Fig. 1a). The peptide sequence with PTMs and charge states are taken as input. The embedding layer contains amino acid sequence embedding, charge state embedding, PTMs position embedding, and PTMs type embedding. The BERT is a pre-trained deep learning model based on Transformer^27^ popular in natural language processing (NLP). As peptide sequence holds similar properties as the natural language, DeepFLR leverages BERT^26^ to learn the representation of phosphopeptides and directly predicts the corresponding tandem mass spectra. We embedded input phosphopeptide sequences into hidden space and then used the BERT encoder to extract interactions among all the amino acid residues, followed with transforming hidden states into outputs with the output layer. Details of the model architecture are explained in the Methods section.

The deep learning model was trained with fourteen higher-energy collisional dissociation (HCD) phosphoproteome datasets (Train_1) with >467,000 peptide-spectrum matches (PSMs) of >120,000 mono-phosphorylated peptides, >63,000 multi-phosphorylated peptides and >165,000 non-phosphorylated peptides for model training (Supplementary Table 1). About 10% of the datasets were left as the validation dataset to verify the increase of MS/MS prediction accuracy over training epochs, minimizing the risk of overfitting to the training dataset. The splitting of the dataset for training and validation was performed without data leakage as detailed in the Methods section. As shown in Supplementary Fig. 1, compared with Transformer, BERT-based model converged faster during training for the MS/MS spectra prediction task. BERT took fewer epochs to reach the plateau value, and to a value higher than the Transformer using the same hyperparameters. Pre-trained on large-corpus, BERT has great initial solution space for downstream tasks so it costs short training time for the task of MS/MS spectra prediction.

The trained deep learning model based on BERT achieved excellent MS/MS prediction performance on the validation dataset, with the median cosine similarity between the experimental and predicted spectra of 0.957 and 0.931 respectively for monophosphopeptides and multiphosphopeptides (Supplementary Fig. 2). The deep learning model was then applied to five LC-MS/MS datasets (Test_1, Test_2, Test_3, Test_4, and Test_5, Supplementary Table 1), independent of the training datasets, of phosphopeptides from different organisms for performance evaluation. DeepFLR predicted phosphopeptide MS/MS accurately for *H. sapiens*, *M. musculus*, *A. thaliana*, *S. cerevisiae* and *E. coli*, with the median cosine similarity between the predicted and experimental mass spectra for mono- and multi-phosphopeptides of all the datasets over 0.86 (Fig. 1b and Supplementary Fig. 3). The results demonstrated that DeepFLR can achieve excellent performance on MS/MS prediction for phosphopeptides from different organisms although it was originally trained with datasets from *H. sapiens*.

To handle the data shift between datasets, fine-tuning was adopted to further enhance the performance of the MS/MS prediction. For each dataset, part of the data was used for fine-tuning and the other was used for testing (see details in Supplementary Table 1). Non-phosphorylated peptides were also included in the fine-tuning, but not for the test. The splitting of the dataset for fine-tuning and testing was performed without data leakage as detailed in the Methods section. It was found that the prediction accuracy was significantly enhanced after fine-tuning, with the median cosine similarity of 0.943 for the *H. sapiens* dataset, 0.955 for the *M. musculus* dataset, and 0.929 for the *A. thaliana* dataset (Fig. 1b). We did not differentiate the mono- and multi-phosphorylated peptides for the cosine similarity calculation after fine-tuning. We also calculated the cosine similarity of repeatedly collected mass spectra of the same phosphopeptides in one dataset to quantify technical variability. It showed that the DeepFLR after fine-tuning can predict phosphopeptide MS/MS at a quality highly close to the experimental replication (Fig. 1b). For instance, the median cosine similarity for the *H. sapiens* dataset by the fine-tuned DeepFLR was 0.943, while it was 0.945 by the experimental replication.

We then compared the performance of DeepFLR in MS/MS prediction with pDeep2^22^ and DeepPhospho^23^ (Figs. 1c and 1d). The results showed that DeepFLR reached higher median cosine similarity for all the test datasets of different organisms than pDeep2 and DeepPhospho. As the performance of DeepPhospho can be enhanced by fine-tuning, we fine-tuned DeepPhospho with different epochs as suggested by the publication of DeepPhospho, using part of the data in Test_1, Test_2, and Test_3 (Supplementary Table 1, Supplementary Figs. 4 and 1c). It turned out that the performance of fine-tuned DeepPhospho still fell back to DeepFLR. We also fine-tuned DeepPhospho using the same training dataset Train_1 of DeepFLR. As a result, the performance of DeepPhospho was enhanced but still not as good as that of DeepFLR (Supplementary Fig. 5). For pDeep2, different normalized collision energy (NCE) parameters ranging from 0.20 to 0.40 were tested (Supplementary Fig. 6), and the performance of MS/MS prediction by pDeep2 with the optimal NCE was still not as good as that of DeepFLR. Besides cosine similarity, we also show the distribution of the Pearson correlation coefficient computed between the predicted and experimental spectra for DeepFLR, DeepPhospho, and pDeep2, respectively (Supplementary Fig. 7).

### Benchmarking the FLR control by DeepFLR on synthetic phosphopeptides datasets

**Fig. 2:**
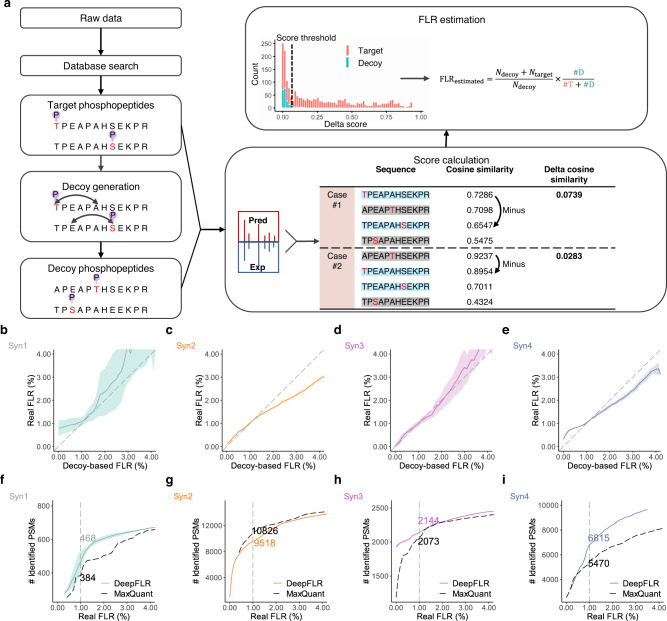
FLR control by DeepFLR. **a** Schematic illustration of the FLR control by DeepFLR. Target phosphopeptides are generated from database searching results by considering all possible phosphopeptides isoforms. Decoy phosphopeptides are generated from the target phosphopeptides by randomly exchanging the phosphorylated S/T/Y with another amino acid residue in the target sequence. MS/MS spectra are predicted by DeepFLR for both target and decoy. Identification and FLR estimation are performed based on spectral matching between experimental spectra and the predicted spectra of both the target and decoy. *N*_decoy_ is the total number of decoy in the database; *N*_*t*arget_ is the total number of target in the database; #*D* is the number of identified decoy hits; and #T is the number of identified target hits. **b–e** Estimated FLR plotted against the real FLR with 95% confidence interval on four synthetic phosphopeptides datasets. The gray dashed line indicates the line where the estimated FLR is equal to the real FLR. **f**–**i** Comparison of the number of identified phosphopeptides PSMs under a given real FLR by DeepFLR in combination with MaxQuant with 95% confidence interval and by MaxQuant alone on the four synthetic phosphopeptides datasets. The numbers of identified PSMs at 1% real FLR are indicated. The colored solid line indicates the mean value of real FLR or identified PSMs, and the shaded area around the solid line indicates the 95% confidence interval of real FLR or identified PSMs. Source data are provided as a Source Data file.

With the accurate prediction of phosphopeptides MS/MS by deep learning, a target-decoy method was established to control the FLR in phosphoproteomics. We tested two different strategies in decoy generation: Method 1, exchanging the whole phosphorylated residue with another non-candidate amino acid residue in the target sequence; and Method 2, randomly shifting the phosphate group to another non-candidate amino acid residue in the target sequence (Supplementary Fig. 8a). The initial peptides list was obtained by protein sequence database searching of the raw LC-MS/MS data, which can be done by different database searching software solutions, e.g. MaxQuant, PEAKS and SpectroMine. Then, the MS/MS of identified phosphopeptides with candidate phosphosites number > the phosphate groups number were extracted for re-analysis by DeepFLR. A target phosphopeptides list was generated from the search result by adding all possible phosphopeptides isoforms, i.e. the combination of all possible phosphates locations on a peptide, and then the decoy for each target was generated using the aforementioned two methods, respectively. Next, MS/MS spectra for both target and decoy phosphopeptides were predicted by the deep learning model. Cosine similarity was calculated between the predicted target/decoy MS/MS and the experimental MS/MS. The top match was reported for each experimental MS/MS with a delta score as the difference value between the maximum cosine similarity and the cosine similarity of a target phosphopeptide closest to the maximum cosine similarity. Identification of all extracted experimental MS/MS was ranked by the delta score, and the FLR was assessed by the presence of target and decoy phosphopeptides in the identification list under a delta score threshold (Fig. 2a). We have chosen four synthetic phosphopeptides datasets (Supplementary Table 2) to evaluate the FLR control using the two decoy generation methods. Based on the synthetic phosphopeptides sequence, empirical or real FLR can be calculated for comparison. As shown in Supplementary Fig. 8b–i, the decoy generation Method 1 can lead to a more accurate estimation of FLR and more identification of phosphopeptides under a given real FLR on all four synthetic phosphopeptides datasets. Therefore, Method 1 was chosen for subsequent data analysis.

We firstly combined the DeepFLR with MaxQuant. The initial phosphopeptide list was from the database searching result by MaxQuant with FDR ≤ 1% and phosphosite probability score ≥ 0. The corresponding MS/MS spectra were then reanalyzed by DeepFLR. The performance of DeepFLR and MaxQuant was assessed on the four synthetic phosphopeptides datasets including three orbitrap datasets (Syn_1, Syn_2, Syn_3) and one quadrupole time-of-flight (Q-TOF) dataset (Syn_4). As the decoy generation can introduce randomicity, we repeated the analysis on the four synthetic phosphopeptides datasets ten times (*n* = 10) to calculate the 95% confidence interval. As shown in Fig. 2b–e and Supplementary Figure 9, on all the four synthetic peptides dataset, the estimated FLR correlated well with the real FLR in a wide range, and a good agreement between the estimated and real FLR was obtained around 1% FLR.

We then compared the number of identified phosphopeptides PSMs at 1% real FLR by MaxQuant and DeepFLR, excluding the ones without phosphopeptide isoforms, i.e. the number of candidate phosphosites = the number of phosphate groups. For Syn_1 (Supplementary Table 2), at 1% real FLR, MaxQuant alone got 384 PSMs and DeepFLR in combination with MaxQuant got 468 PSMs on average (Fig. 2f). Since Syn_1 is very small (130 phosphopeptides, Supplementary Table 2), a relatively large variation in the number of PSMs was observed. For all the 10 tests, DeepFLR got more PSMs than MaxQuant alone. It should be noted that the MaxQuant localization probability score was 0.99 to achieve the 1% real FLR on this dataset, in contrast to the recommended value of 0.75^28^.

Then, we compared DeepFLR and MaxQuant on another larger synthetic phosphopeptide dataset acquired on an LTQ Orbitrap Velos mass spectrometer. As the pre-trained DeepFLR deep learning model was based on data from the newer version of Orbitrap mass spectrometers, i.e. Q Exactive, Q Exactive HF, Q Exactive Plus, and Orbitrap Fusion, fine-tuning was applied for this dataset using about half of the raw files (Supplementary Table 2 and Methods section). The refined DeepFLR model was then used to identify phosphopeptides from the rest raw files. The splitting of the dataset for fine-tunning and test was performed without data leakage as detailed in the Methods section. At 1% real FLR, MaxQuant alone got 10,826 PSMs (localization probability of 0.98), and DeepFLR in combination with MaxQuant got 9518 PSMs on average (Fig. 2g). To note, the 95% confidence interval decreased with sample size increasing. Herein, less PSMs were identified by DeepFLR compared to MaxQuant. As noted by another publication and the original paper, due to the synthesis strategy adopted by the work, there can be numerous peptides with similar sequences in the dataset^29,30^, which may limit the data analysis performance of the target-decoy approach adopted by DeepFLR.

DeepFLR can be further extended to multiphosphorylated peptides (Supplementary Fig. 10). For decoy generation, each of the phosphorylated residues was exchanged randomly with another non-candidate amino acid residue in the peptide sequence. For one target double-phosphorylated peptide, two decoys can be generated. The FLR calculation remains unchanged. We tested the method on the synthetic phosphopeptides dataset Syn_3 (Supplementary Table 2), which includes multi-phosphorylated and mono-phosphorylated peptides. At 1% real FLR, MaxQuant alone identified 2073 PSMs (localization probability of 0.83), and DeepFLR in combination with MaxQuant identified 2144 PSMs on average (Fig. 2h). The identification results include both mono- and multi-phosphorylated peptides.

To demonstrate the performance of DeepFLR on other types of instruments, the synthetic phosphopeptides dataset Syn_4 acquired by Q-TOF was further adopted (Supplementary Table 2). Since Q-TOF is significantly different from orbitrap instrument, re-training of the DeepFLR deep learning model was applied using three Q-TOF datasets containing 34,077 PSMs of 16,297 phosphopeptides and 12,471 non-phosphorylated peptides (Train_2, Supplementary Table 2). About 10% of the datasets were left as the validation dataset. The re-trained DeepFLR achieved good MS/MS prediction performance with the median cosine similarity against experimental MS/MS of 0.84 and 0.87 on the Syn_4 and the validation dataset of Train_2, respectively (Supplementary Table 2, Supplementary Fig. 11). With the re-trained deep learning model, DeepFLR in combination with MaxQuant localized 24.6% phosphosites (6815 on average) more than MaxQuant alone (5470) at 1% real FLR (Fig. 2i). For MaxQuant, the localization probability was 0.90 to get 1% real FLR.

DeepFLR can also be combined with other database searching tools, such as SpectroMine^31^ and PEAKS^32^. With Syn_3, DeepFLR combined with SpectroMine obtained 2907 phosphopeptides PSMs at 1% real FLR, while in contrast the SpectroMine alone cannot reach 1% FLR by optimizing the localization probability score (P.LocalizationConfidence), Supplementary Fig. 12. Combining with PEAKS, DeepFLR identified 1832 phosphopeptides PSMs at 1% real FLR, while PEAKS alone identified 1813 phosphopeptides PSMs at 1% real FLR where the localization probability score (AScore) was 10.2 instead of the recommended AScore of 20^7^, Supplementary Fig. 12. Combining with either SpectroMine or PEAKS, DeepFLR estimated accurately the FLR for Syn_3 (Supplementary Fig. 12). It should be noted that DeepFLR reanalyzes the results of database searching for phosphopeptides identification, and hence its performance is related to the software solutions used for database searching. For Syn_3, DeepFLR obtained the most PSMs when combining with SpectroMine.

We further compared DeepFLR with recently published localizing algorithm AscorePro^33^ and PhosphoRS^10^. DeepFLR outperformed AscorePro and PhosphoRS in sensitivity (Supplementary Fig. 12). We also compared DeepFLR to other FLR control method, i.e. LuciPHOr2^34^, the extended version of LuciPHOr^5^, with Syn_3 based on the database searching result by MaxQuant. LuciPHOr2^34^ failed to filter wrong hits and the real FLR only dropped to 4% when the estimated FLR reached 0 (Supplementary Fig. 12). In contrast, DeepFLR can estimate accurately the FLR and identified 2144 PSMs at 1% real FLR (Fig. 2h, Supplementary Fig. 12).

To illustrate the performance of the target-decoy approach, the delta score distribution of the top hit of each MS/MS for all the synthetic datasets was plotted. As shown in Supplementary Fig. 13, the distribution of decoy hits was similar to that of false hits. Therefore, the decoy hits can trace how false hits distribute, and thus the DeepFLR can estimate the real FLR. In contrast, the median of the delta score of true hits was much larger than that of the decoy or false hits. Hence, most true identification results can be retained during the FLR control to guarantee good detection sensitivity. A bi-modal behavior can be observed for the false hits on some synthetic datasets, which can be due to the small size of the datasets that can lead to randomicity and permutation. With the largest synthetic dataset, Syn_2, there is no obvious bi-modal behavior.

To exemplify how the DeepFLR can localize a phosphorylation site, the identification of a MS/MS for the phosphopeptide pSTLVLHDLLK is illustrated in Supplementary Fig. 14. The phosphopeptide has two adjacent potential phosphorylation sites, S1 and T2. The site-determining ions are b1 and y9, which are missed in the MS/MS spectra, making the two sites not distinguishable by database searching tools based on the observation of site-determine ions. In DeepFLR, the MS/MS was successfully identified as pSTLVLHDLLK, with a delta score of 0.080. This spectrum was from the Syn_1 dataset, where a delta score threshold of 0.054 corresponded to the FLR of 0.01. Therefore, DeepFLR can benefit the identification of phosphopeptides without site-determining ions.

### Analysis of biological samples by DeepFLR

After assessing the performance of DeepFLR on the synthetic phosphopeptides datasets, DeepFLR was further applied to large-scale biological sample datasets. As the MS/MS prediction accuracy can be enhanced through transfer learning, fine-tuning was applied to DeepFLR before the analysis of the biological sample datasets (see details in the Methods section). Although DeepFLR can handle phosphopeptides with more than two phosphate groups, multiphosphopeptides beyond doubly phosphorylated peptides are less frequently observed and hence discarded in these examples to save computation time. Based on the evaluation using synthetic phosphopeptides datasets, the MaxQuant localization probability threshold was set as 0.99 for 1% FLR. The phosphopeptides identified by MaxQuant without phosphopeptides isoforms, i.e. the number of candidate phosphosites = the number of phosphate groups, were kept in the identification list of both DeepFLR and MaxQuant for comprehensive bioinformatic analysis.

**Fig. 3:**
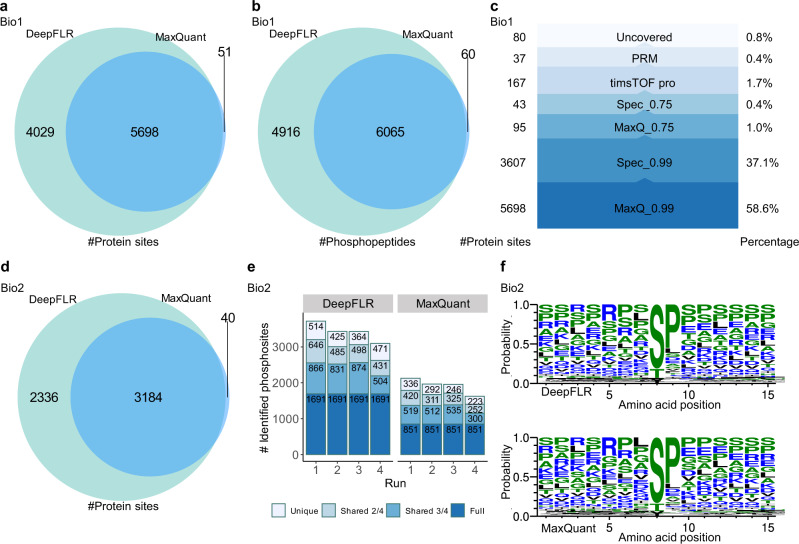
Analysis of biological samples Bio_1 and Bio_2 by DeepFLR. Venn diagram of the number of **a** protein phosphosites and **b** phosphopeptides identified by DeepFLR and MaxQuant for Bio_1. **c** Stepwise coverage of the DeepFLR localized phosphosites. The height of the bar chart is proportional to the log value of the number of additionally localized protein phosphosites. MaxQ_0.99 (0.75): MaxQuant with 0.99 (0.75) localization probability; Spec_0.99 (0.75): SpectroMine with 0.99 (0.75) localization probability. PRM: parallel reaction monitoring. **d** Venn diagram of the number of phosphosites identified by DeepFLR and MaxQuant for Bio_2. **e** Histogram of the number of phosphosites identified among the technical replicates by DeepFLR and MaxQuant. Full represents identifications observed in all the 4 runs; Shared 3/4 represents identifications observed in 3 runs; Shared 2/4 represents identifications observed in 2 runs; Unique represents identifications observed in only 1 run. **f** Amino acid sequence logo for identified phosphosites. Each column of the alignment is represented by a stack of letters where the height of each letter is proportional to the normalized frequency of the corresponding amino acid.

We collected the first benchmark biological dataset Bio_1 using an Orbitrap Fusion Lumos Tribrid mass spectrometer. The sample was phosphopeptides enriched from the tryptic digests of proteins from Hela cells without any specific treatment (Supplementary Table 3, Methods section). At 1% estimated FLR, DeepFLR combining MaxQuant localized 9727 phosphosites, while MaxQuant alone localized 5749 phosphosites with 0.99 localization probability (Fig. 3a, Supplementary data 1). DeepFLR covered 99% of the phosphosites localized by MaxQuant alone. On the phosphopeptides level, DeepFLR identified 4856 phosphopeptides more than MaxQuant alone and covered 99% of the phosphopeptides identified by MaxQuant alone (Fig. 3b, Supplementary data 1). To test the reliability of the 9727 protein phosphosites localized by DeepFLR, stepwise coverage was applied (Fig. 3c). For all the 9727 phosphosites, 5698 proteins phosphosites were covered by MaxQuant with 0.99 localization probability. For the rest 4029 phosphosites, 3607 were covered by SpectroMine analysis of the same dataset with 0.99 localization probability. Furthermore, 95 remaining phosphosites were covered by MaxQuant with 0.75 localization probability, and then 43 by SpectroMine with 0.75 localization probability. We have also analyzed the same sample by another mass spectrometer, the timsTOF Pro2, using the parallel accumulation serial fragmentation data dependent acquisition (PASEF-DDA) strategy, and analyzed the data by SpectroMine with 0.75 localization probability, where 167 additional protein phosphosites were identified. Finally, the remaining uncovered phosphosites were subjected to targeted analysis. We checked the MS/MS spectra manually and identified another 37 protein phosphosites with the support of site-determining fragment ions (Supplementary data 2). Finally, there were only 80 (0.8%) protein phosphosites identified by DeepFLR not covered by any other software solutions or analysis methods adopted here. The results supported the reliability of the DeepFLR in phosphopeptides identification.

The second benchmark biological dataset is from the phosphopeptides enriched from the tryptic digests of proteins of MCF7 breast cancer cells treated with a cocktail of insulin-like growth factor-1, epidermal growth factor and pervanadate, originally published by Lawrence et al. (PXD003344, Bio_2 Supplementary Table 3)^35^. At 1% estimated FLR, DeepFLR combining MaxQuant localized 5520 protein phosphosites (Fig. 3d) compared to 3224 by MaxQuant alone with 0.99 localization probability (Supplementary data 3). For 2336 additionally localized protein phosphosites by DeepFLR, 1593 were reported by the original publication from the same sample combining the results by different mass spectrometry methods^35^ (Supplementary Fig. 15a). It should be noted that more kinase-regulated sites were identified by DeepFLR than MaxQuant alone (Supplementary Fig. 15b). At the phosphopeptides level and PSMs level, DeepFLR also gave more identifications than MaxQuant alone (Supplementary Fig. 15c, d). By comparing the identification results from technical replicates, it was found that 30.6% protein phosphosites were identified from all four technical replicates by DeepFLR, while only 26.4% by MaxQuant alone (Fig. 3e), demonstrating that DeepFLR can provide identification results with higher consistency among replication. Similar results were observed at the phosphopeptides level (Supplementary Fig. 15e). Then, the sequences of phosphopeptides identified by DeepFLR and MaxQuant alone were exploited to construct a sequence logo (Fig. 3f), where the 7 amino acid residues before and after the phosphorylated S/T/Y were displayed. It showed that the phosphopeptides identified by DeepFLR and MaxQuant shared a similar pattern of sequence logo, but the frequency of threonine slightly rose while tyrosine was almost unchanged and serine dropped in the DeepFLR-based result, indicating that DeepFLR has a stronger capability to identify low-abundant phosphothreonines. Revealed by the sequence logo results, the prevalence of proline at position n + 1 may act as the sign of proline-directed kinases^36^.

**Fig. 4:**
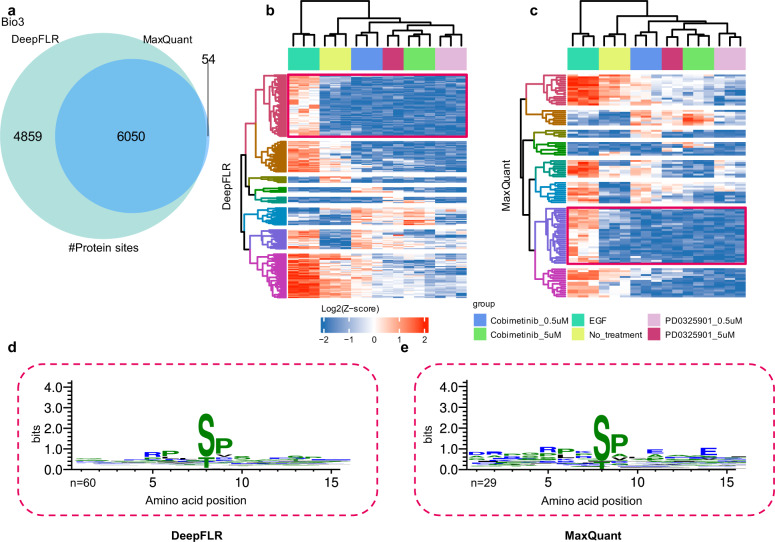
Analysis of biological sample Bio_3 by DeepFLR. **a** Venn diagram of the number of protein phosphosites identified by DeepFLR and MaxQuant alone. Heatmap of unsupervised clustering analysis of significantly regulated phosphosites obtained by **b** DeepFLR and **c** MaxQuant alone. Amino acid sequence logo for the EGF up-regulated and MEK inhibitors down-regulated phosphosites localized by **d** DeepFLR and **e** MaxQuant (the ones in the red rectangles). Each column of the alignment is represented by a stack of letters where the height of each letter is proportional to the normalized frequency of the corresponding amino acid. The numbers of phosphosites (*n*) used are indicated below each graph. Source data are provided as a Source Data file.

The third benchmark biological dataset is from a study by Bekker-Jensen et al. (PXD014525, Bio_3, Supplementary Table 3)^37^. The dataset is based on epidermal growth factor (EGF)-stimulated retinal pigment epithelium (RPE1) cells with six conditions of no treatment, EGF treated only, and EGF treated with MEK inhibitors of Cobimetinib (5 μM or 0.5 μM) or PD0325901 (5 μM or 0.5 μM). At 1% estimated FLR, DeepFLR combining MaxQuant identified 10,909 protein phosphosites, compared to 6104 by MaxQuant alone with 0.99 localization probability (Fig. 4a, Supplementary data 4). For the 4859 DeepFLR additionally localized protein phosphosites, 3898 were reported by the original paper^37^ combing all the results of different mass spectrometry methods for the same sample (Supplementary Fig. 16a). Better performance by DeepFLR was also observed at the phosphopeptides and PSMs level (Supplementary Fig. 16b, c). To reveal the new biological insights by DeepFLR, we performed an analysis of variance (ANOVA) test to identify significantly regulated sites. DeepFLR identified 186 significantly regulated phosphorylation sites, nearly twice as many as the number of significantly regulated sites identified by MaxQuant alone (99) (Supplementary data 5). Unsupervised hierarchical clustering was performed to generate a heatmap of the significantly regulated phosphosites obtained by DeepFLR (Fig. 4b) and MaxQuant (Fig. 4c). Similar patterns of regulated phosphorylation events revealed by the two tools were observed. The red rectangles highlight the clusters of phosphosites stimulated by EGF and regulated by MEK. The EGF treatment induces the phosphorylation of its receptor^38^, and in turn activates downstream mitogen-activated protein kinase (MAPK) pathway, which involves the activation of extracellular signal-regulated kinase (MEK1 and MEK2), and MAPK (ERK2 and ERK1) kinases^39^. MEK1/2 regulate ERK, while ERK regulates a wide variety of cellular functions by phosphorylating its substrates including p90RSK1, MNK1, MNK2, and TOB^38,40^. The MEK inhibitors added after EGF stimulation can inactivate the phosphorylation of ERK and hence down-regulate the phosphorylation of the substrates of ERK^41^. The phosphosites of the cluster highlighted by the red rectangles were exploited to construct sequence logos for DeepFLR (Fig. 4d) and MaxQuant (Fig. 4e). Both sequence logos demonstrated a Px[s/t]P motif (Fig. 4d, e), which is in accordance to the known ERK kinase substrate motif (Supplementary Fig. 16d), demonstrating the biologically relevant information from the additionally identified phosphosites by DeepFLR. It should be noted that although the sequence logos of DeepFLR and MaxQuant alone showed similar pattern, DeepFLR localized ERK substrate phosphosites (*n* = 60) more than that by MaxQuant alone (*n* = 29).

It should be noted that the significantly enhanced number of identified phosphosites by DeepFLR compared to MaxQuant on the biological samples can be a result of the high localization probability threshold we set for MaxQuant for a conservative estimate. To provide a more comprehensive comparison, we show in Supplementary Figure 17 how the number of phosphosites changes as the MaxQuant localization probability cutoff is varied from 0.75 to 1 for the three biological datasets Bio_1, Bio_2, and Bio_3. With the decrease of MaxQuant probability cutoff, the number of phosphosites increases significantly. Nevertheless, even at the probability cutoff of 0.75, the number of localized phosphosites by MaxQuant alone are fewer than that by DeepFLR in combination with MaxQuant at 1% estimated FLR on all three biological datasets, demonstrating the good performance of DeepFLR.

### Phosphopeptides DIA analysis with spectral libraries built by DeepFLR

Recently, DIA has also been used for phosphoproteomics^37^. In contrast to DDA, where the MS/MS is acquired based on the observation of precursor ions, DIA performs a sequence of MS/MS scans within defined isolation windows in each acquisition cycle, recording fragmentation information of all peptides in a sample^42^. The DIA data can be analyzed by the spectrum-centric and peptide-centric strategies. In the spectrum-centric strategies, pseudo MS/MS spectra are generated by assembling precursor-fragment groups based on the elution profiles of precursor and fragment ions, and then subjected to routine DDA database search. In the peptide-centric strategies, target peptides are queried against DIA data to extract the best candidate chromatogram signals using prebuilt spectral libraries by DDA analysis of the same sample containing the information of retention time and fragment ions. The peptide-centric strategies can normally lead to more sensitive identification.

**Fig. 5:**
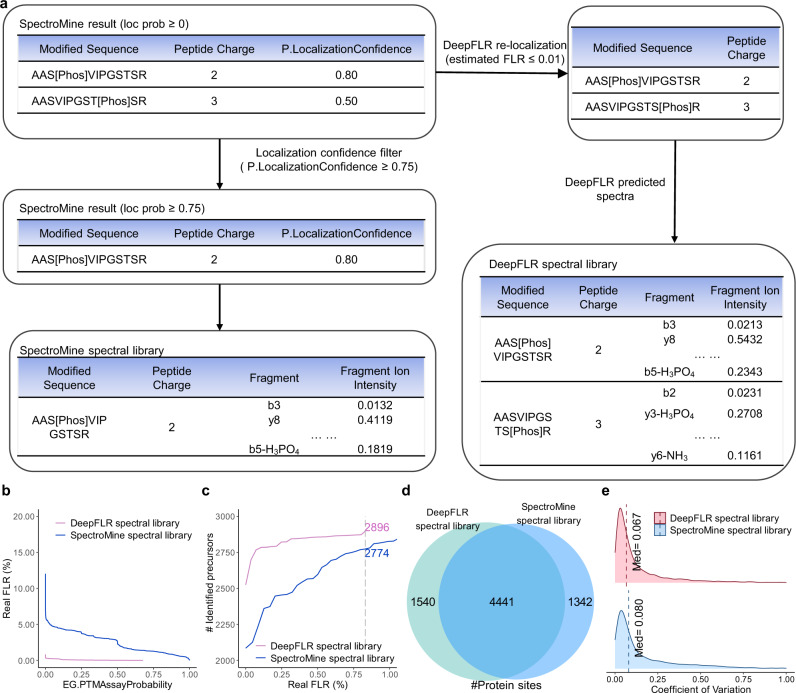
DIA analysis using DDA-based experimental spectral library and predicted spectral library by DeepFLR. **a** Schematic illustration of the spectral libraries construction. Identification of phosphopeptides from a synthetic phosphopeptides dataset DIA_1 using DeepFLR predicted spectral library and SpectroMine-based experimental spectral library, considering **b** the correlation between real FLR and EG.PTMAssayProbability score by Spectronaut analysis of the DIA data, and **c** the correlation between real FLR and identified phosphopeptides precursors. **d** Venn diagram of the number of phosphosites localized for a biological phosphopeptides dataset DIA_2 using DeepFLR predicted spectral library and SpectroMine-based experimental spectral library. **e** Coefficient of variation calculated based on the protein phosphosites quantity among three technical replicates in DIA_2 using DeepFLR predicted spectral library and SpectroMine-based experimental spectral library. Source data are provided as a Source Data file.

In peptide-centric DIA analysis, the quality of the spectral library is crucial for subsequent DIA analysis. As show in Supplementary Fig. 18, when the spectral library contains a large amount of phosphopeptides with incorrect phosphosites, detection sensitivity is significantly restricted. Herein, we used DeepFLR to generate spectral libraries with accurate phosphosites to facilitate DIA analysis. DDA data for spectral library building was firstly processed by SpectroMine with localization probability threshold of 0, and the MS/MS of identified phosphopeptides were re-analyzed by DeepFLR for phosphopeptides identification with an estimated FLR of 0.01. Three spectral libraries were generated by DeepFLR for DIA data analysis, including the predicted spectral library (Fig. 5a), the hybrid spectral library and the re-localized experimental spectral library. For the predicted spectral library, all MS/MS spectra were predicted by DeepFLR. For the hybrid spectral library, if DeepFLR identified a different phosphopeptide compared to SpectroMine from a MS/MS spectrum, the MS/MS spectrum of the phosphopeptide in the library was generated by DeepFLR prediction, while the others were from DDa experiment. For the re-localized experimental spectral library, all MS/MS spectra were from the experiment but the phosphopeptide identification result was from DeepFLR. All the retention time values in all three libraries were from experiment. For comparison, DDA-based experimental spectral library was also generated by SpectroMine with the localization probability threshold of 0.75 (Fig. 5a). We tested the performance of the spectral libraries on a synthetic phosphopeptides DIA dataset (DIA_1, PXD014525^37^) (Supplementary Table 4). As shown in Supplementary Figure 19 and Fig. 5b, c, the predicted spectral library led to the largest number of identified phosphopeptides precursors among all the three libraries. Precursors from different DIA runs were count separately. Even without localization filtration during the DIA data analysis by Spectronaut, the real FLR was smaller than 1% (0.83%) when using the DeepFLR predicted spectral library. In contrast, the EG.PTMassayprobability needed to reach 0.84 during the DIA data analysis to reduce the real FLR to 0.01 and 0.88 to reduce the real FLR to 0.83% when using the SpectroMine spectral library (Fig. 5b**)**. For 0.83% real FLR, DIA search results based on the DeepFLR predicted spectral library identified 2896 phosphopeptides precursors, while the DIA search result based on the SpectroMine spectral library identified only 2774 phosphopeptides precursors (Fig. 5c). We also compared the performance of DeepPhospho with DeepFLR. As shown in Supplementary Fig. 20, DeepFLR outperforms DeepPhospho in both sensitivity and localization accuracy. For the DeepPhospho predicted spectral library, the phosphopeptides in the library were obtained by SpectroMine analysis of the corresponding DDA datasets with 0.75 localization probability threshold. Both the MS/MS spectra and the retention time values were predicted by DeepPhospho.

The performance of the predicted spectral library by DeepFLR was further tested on a biological sample dataset originally published by Searle et al. (DIA_2, MSV000082956^43^, Supplementary Table 4). The deep learning model of DeepFLR was fine-tuned by part of the DDA data corresponding to the DIA data (Supplementary Table 4, Methods). The EG.PTMassayprobability cutoff was set as 0 and 0.88 for the analysis using the DeepFLR predicted spectral library and the SpectroMine-based experimental spectral library, respectively, based on the results from the synthetic phosphopeptides dataset DIA_1. 5981 protein phosphosites were localized using the DeepFLR predicted spectral library, while 5783 were localized by using the SpectroMine spectral library, with 4441 shared by the two libraries (Fig. 5d, Supplementary data 6). The coefficient of variation (CV) among three technical replicates was calculated for the identified phosphosites. The CV based on the DeepFLR predicted spectral library was smaller than that based on the SpectroMine spectral library (Fig. 5e).

## Discussion

In this study, we propose DeepFLR to address the FLR control issue in phosphoproteomics via a target-decoy approach, where MS/MS of target and decoy phosphopeptides are predicted by deep learning and used for spectra matching-based phosphopeptides identification. The performance of DeepFLR was benchmarked on synthetic phosphopeptides datasets and biological sample datasets acquired by different types of instrument (orbitrap and Q-TOF) and with different data acquisition strategies (DDA and DIA). It should be noted that DeepFLR is only used to locate phosphate groups on peptides and needs to be used with different sequence database searching tools, such as MaxQuant, PEAKS and SpectroMine, for peptide identification. In DIA, DeepFLR is used to build a spectral library based on DDA data database searching results. We have demonstrated that DeepFLR can lead to more identification of phosphosites and accurate FLR control. In the case of biological datasets, the additionally identified phosphosites were verified using different software solutions and different mass spectrometry-based approaches, such as PRM and PASEF-DDA, as well as biological settings. DeepFLR does not restrict precursor charge states, predicts 36 fragment types, handles sequence length less than 512 amino acids, and considers four common PTMs (Phospho (STY), Oxidation (M), Acetyl (Protein N-term), Carbamidomethyl (C)), covering most of the phosphopeptides under normal circumstances. The MS/MS spectra prediction speed is 110 spectra/s using RTX 3090 (GPU) and 20 spectra/s with AMD Ryzen 7 4800U (CPU), which is fast enough for most current phosphoproteomics studies.

The deep learning model of DeepFLR is based on BERT, the bidirectional encoder representations from Transformer. Compared to the existing deep learning models for phosphopeptides MS/MS spectra prediction, i.e. pDeep2^22^ and DeepPhospho^23^, DeepFLR enhances prediction accuracy. Different from the bidirectional LSTM used in pDeep2, BERT used by DeepFLR utilizes attention mechanism that enables each token to interact with all the other tokens whatever distance between them, thus capturing long-range dependency. Compared with DeepPhospho, the versatility of BERT can substitute the Bi-LSTM module in DeepPhospho. BERT is a model pretrained to understand natural language. Considering the fact that peptide sequence holds similar properties as the natural language, we used BERT with pre-knowledge from natural language processing for a better initialization of representation and achieved better prediction performance than the Transformer without pretraining. The utilization of publicly-available pretrained general model is also a common method to reduce training cost^44^.

DeepFLR utilizes a target-decoy strategy for FLR control. The most commonly used decoy generation for FDR control in sequence database searching is the reversal or shuffle of peptide sequences. When the idea of the target-decoy method is extended to spectra searching, decoy spectra are usually generated by manipulation of real spectra or by machine learning-based prediction^45,46^. Nonetheless, the decoy generation approaches for FDR control cannot accurately capture errors associated with partial matches including the incorrect assignment of PTMs sites^46^. For FLR control, LuciPHOr^5^ generates decoys by adding phosphate groups to all the non-candidate amino acid residues. However, adding phosphate groups on all the residues except S/T/Y results in the generation of much more decoys than the targets, which can lead to reduced sensitivity and large computational costs. SLIP score^13^ adds phosphorylated proline or glutamic acid during database searching. As the frequency of amino acids varies among datasets, choosing specific residues to generate decoys can result in distinct performances for different datasets. In this work, we tested two decoy generation approaches, by exchanging the whole phosphorylated amino acid residue with another non-candidate amino acid residue, or by randomly shifting the phosphate group to another non-candidate amino acid residue. The method of exchanging residue can estimate FLR accurately with high sensitivity.

Currently, the most commonly used workflow for phosphoproteomics is based on protein sequence database searching, using software solutions such as MaxQuant^8^, PEAKS^32^, etc., together with built-in probability-based localization algorithms, such as PTM score^9^, AScore^7^, etc. In this study, the original identification result of phosphopeptides is obtained by MaxQuant, PEAKS, or SpectroMine, and DeepFLR is used to re-localize phosphate groups for each identified phosphopeptide and control FLR. DeepFLR achieves excellent performance in combination with all the protein sequence database searching tools. As demonstrated with four synthetic phosphopeptides datasets, the DeepFLR can estimate accurately 1% FLR for the identification of phosphopeptides. In contrast, it is hard to define a universal score threshold to determine FLR using probability-based localization algorithms. For Syn_1, Syn_2, Syn_3, and Syn_4, the MaxQuant probability scores for 1% FLR are 0.99, 0.98, 0.83, and 0.90, respectively. However, a probability score threshold of 0.75 is recommended in many publications^24,28,47^, making the phosphosite localization less reliable in the proteomic study of real biological samples, and hence introducing cumbersome tasks to the following biological validation.

Although DeepFLR can estimate accurately 1% FLR for all the synthetic phosphopeptides datasets, fewer phosphopeptides are identified by DeepFLR than MaxQuant for Syn_2, which is a large synthetic phosphopeptides dataset, acquired by Orbitrap LTQ Velos system. In contrast, DeepFLR identifies more phosphopeptides than MaxQuant for the other synthetic phosphopeptides datasets. One possible reason for the restricted performance for Syn_2 can be due to the synthesis strategy adopted to generate the dataset. The synthetic phosphopeptide library was built from 96 seed peptides^29^, which included numerous peptides with similar sequences and very different from the condition of the real biological samples. The numerous isomers in the library can render the data analysis performance of the target-decoy strategy adopted by DeepFLR. We hope that more synthetic phosphopeptides datasets would be available in the near future, with which the target-decoy strategy for FLR estimation can be further optimized to achieve a more accurate estimation of FLR in a wide range and more sensitive identification of phosphopeptides. To further improve the detection sensitivity, contrastive learning^48^ can be used to increase the differentiation between false phosphosites and correct phosphosites.

DIA is an emerging alternative to DDA to provide comprehensive information on samples. For peptide-centric DIA data analysis, it is important to build a spectral library highly specific to the samples, including as many as possible phosphopeptides with accurate phosphosites information. We tested three spectral libraries construction methods, including the predicted spectral library, the hybrid spectral library, and the re-localized experimental spectral library, among which the predicted spectral library performs the best. The quality of the MS/MS spectra in the predicted library can be better than the experimental MS/MS spectra when the peptides are at low abundance or when there is co-elution of peptides in the isolation window of MS/MS acquisition by DDA, hence leading to better performance in DIA analysis. DeepFLR can also be used to assist the spectrum-centric analysis of DIA data for phosphoproteomics by performing spectra deconvolution using DIA-Umpire^49^.

We anticipate that DeepFLR can be a key metric for FLR control just as the conventional target-decoy measure for FDR in peptide identification. DeepFLR can also be used to validate the localization credibility of datasets and even public knowledgebases to promote downstream usage. Besides, it can evaluate different computational pipelines by classifying the individual score thresholds of a given estimated FLR. We outlook that the concept of DeepFLR can be extended to enhance site localization confidence in other PTMs, such as glycosylation.

## Methods

### Phosphopeptides sample preparation

Human epithelial cervical cancer cells (HeLa, CCL-2, the American Type Culture Collection (ATCC), Manassas, VA, USA) were cultured in Gibco Dulbecco’s Modified Eagle Medium (DMEM) with 10% fetal bovine serum and Penicillin-Streptomycin at 37 °C in an incubator with 5% CO_2_. Cells were harvested at approximately 80% confluency by incubation with 0.25% trypsin/EDTA, washed three times with PBS, pelleted by centrifugation for 5 min at 3000 *g,* and collected in a lysis buffer cocktail (8 M urea,1 mM EDTA, 1 mM CAA, 150 mM NaCl, 50 mM Tris-HCl pH 8.0, protease inhibitor, and phosphatase inhibitors). The samples were then lysed on ice for 5 min and centrifuged at 21,500 *g* for 20 min at 4 °C. Proteins were collected from the supernatant and quantified by using the Pierce™ BCA Protein Assay Kit (Thermo Fisher Scientific, Waltham, MA, USA). Five hundred micrograms of protein were transferred into a new centrifugation tube and the final volume was adjusted to 400 μL with 8 M urea. Ten microlitres of 0.5 M tris(2-carboxyethyl)phosphine (TCEP) was added and the sample was incubated at 37 °C for 1 h. Then 20 μL of 1 M iodoacetamide was added to the sample and the incubation was last for 40 minutes protected from light at room temperature. After that, five volumes of −20 °C pre-chilled acetone were added to precipitate the proteins overnight at −20 °C. The precipitates were washed with 1 mL pre-chilled 90% acetone aqueous solution twice and then re-dissolved in 500 μL 100 mM tetraethylammonium bromide (TEAB). Sequence grade modified trypsin (Promega, Madison, WI, USA) was added at the ratio of 1:50 (enzyme: protein, weight: weight) to digest the proteins at 37 °C overnight. The peptide mixture was desalted by Sep-Pak C18 1 cc Vac Cartridge (WAT054955, Waters, Milford, MA, USA), quantified by Pierce quantitative colorimetric peptide assay (Thermo Fisher Scientific, Waltham, MA, USA), and then lyophilized by SpeedVac (Waters, Milford, MA, USA). The phosphopeptides were enriched with High-Select^TM^ TiO_2_ Phosphopeptide Enrichment Kit (Thermo Fisher Scientific, Waltham, MA, USA) following the manufacturer’s instructions. The enriched phosphopeptides were dried by a speed vacuum concentrator.

### LC-MS/MS analysis

The phosphopeptides enriched from 1 mg of peptides were re-dissolved in 15 μL buffer A (0.1% formic acid in water) and analyzed by Orbitrap Fusion Lumos coupled to an EASY-nanoLC 1200 system (Thermo Fisher Scientific, Waltham, MA, USA) with Thermo Xcalibur (version 3.0.63) for data collection. A total of 4 μL peptide sample was loaded onto a 25 cm analytical column (75 μm inner diameter, 1.9 μm resin, Dr. Maisch) and separated with a 120 min-gradient starting at 4% buffer B (80% ACN with 0.1% FA) for 4 min followed by a stepwise increase to 50% in 116 min, 95% in 1 min and stayed there for 9 min. The column flow rate was maintained at 250 nL/min with a column temperature of 55 °C. The electrospray voltage was set to 2 kV. The mass spectrometer was run under data-dependent acquisition (DDA) mode, and automatically switched between MS and MS/MS mode. The survey of full scan MS spectra (m/z 350–1500) was acquired in the Orbitrap with 1,200,000 MS resolution. The automatic gain control (AGC) was targeted at 4e5 and the maximum injection time was 50 ms. Then the precursor ions were selected into the collision cell for fragmentation by HCD, the collision energy was 30. The MS/MS resolution was set at 30,000, the automatic gain control (AGC) targeted at 10^5^, the maximum injection time was 540 ms, the isolation window was 4 m/z, and dynamic exclusion was 30 seconds.

The same sample was also analyzed by timsTOF pro2 (Bruker Daltonics, Bremen, Germany) with Bruker Compass HyStar 6.0 for data collection. The UltiMate 3000 (Thermo Fisher Scientific, Waltham, MA, USA) LC system was connected to the timsTOF Pro2. Samples enriched from 0.5 mg of peptides were re-dissolved in 20 μL 0.1% FA (buffer A), and 4 μL was separated by an analytical column (25 cm × 75 μm i.d., Evosep) with a 72 min gradient starting at 4% buffer B (80% ACN with 0.1% FA) followed by a stepwise increase to 23% in 50 min, 44% in 10 min, 90% in 7 min and stayed there for 5 min. The column flow rate was maintained at 300 nL/min with a column temperature of 50 °C. The instrument was operated in the PASEF-DDA mode with 10 PASEF scans per topN acquisition cycle and accumulation and ramp times of 100 ms each. MS and MS/MS spectra were recorded from 100 to 1700 m/z and an ion mobility range (1/K0) of 0.6–1.6 Versus/cm^2^ was used. Including charge was set to 0–5, the target value was set to 10,000 and dynamic exclusion was activated and set to 0.4 min. The quadrupole isolation width was set to 2 Th for m/z < 700 and 3 Th for m/z > 700.

The PRM analysis was performed using the EASY-nanoLC 1200 coupled Orbitrap Fusion Lumos system with Thermo Xcalibur (version 3.0.63) for data collection. The sample amount and LC condition were same as the one for DDA analysis. PRM settings were as follow: Full MS scans in the mass range from m/z 350 to 1500 were acquired with a resolution of 120,000, normalized AGC target of 200% and a maximum injection time of 50 ms. MS2 spectra were acquired with a resolution of 30,000, a normalized AGC target of 200%, and a maximum injection time of 50 ms. The inclusion list was imported into the mass list table in the PRM mode.

### Phosphoproteome data collected from public resources

The datasets and raw files used have been summarized in Supplementary Data 7. In general, data was split by embedding pattern (precursor charge, PTMs position, PTMs type, and peptide sequence) for training/validation during model training or fine-tuning. If there were spectra corresponding to the same combination of embedding patterns, only the spectrum with the highest PSM score was retained.

For deep learning model training, 14 raw datasets (Train_1, Supplementary Table 1) from PRIDE^50^ were obtained with the accession number PXD004452^51^, PXD001374^52^, PXD001305^53^, PXD003529^54^, PXD002135^55^, PXD004447^56^, PXD000612^57^, PXD001565^58^, PXD004252^59^, PXD001550^60^, PXD001546^60^, PXD002286^61^, PXD003531^62^ and PXD002394^63^. The combined datasets were divided into the training dataset and the validation dataset with a ratio of about 9:1. The training datasets contain 349,816 peptides, including 120,450 singly phosphorylated peptides and 63,580 multiply phosphorylated peptides and 165,786 non-phosphopeptides. The validation datasets contain 50,331 peptides, including 17,491 singly phosphorylated peptides, 9090 multiply phosphorylated peptides, and 23,750 non-phosphopeptides. All the spectra had different embedding patterns. There was no data leakage during the model training.

For model re-training to fit the Q-TOF data, 3 datasets (Train_2, Supplementary Table 2) from PRIDE^50^ were obtained with the accession number PXD006056^64^, PXD012433^65^, and PXD015687^66^. The combined datasets were also divided into the training dataset and the validation dataset with a ratio of about 9:1. The training datasets contain 34,077 PSMs of 16,297 phosphopeptides and 12,471 non-phosphorylated peptides. The validation datasets contain 3786 PSMs of 2166 phosphopeptides and 1550 non-phosphorylated peptides. All the spectra had different embedding patterns.

To evaluate the performance of DeepFLR in MS/MS spectra prediction, five datasets were downloaded from PRIDE^50^ with the accession number PXD018663^67^ (Test_1), PXD019697^68^ (Test_2), PXD011284^69^ (Test_3), PXD023361^70^ (Test_4) and PXD008211^71^ (Test_5). The data are from five organisms, containing 12,968 (*H. sapiens)*, 22,437 (*M. musculus)*, 2674 (*A. thaliana)*, 136 (*S. cerevisiae)*, and 69 (E. coli) phosphopeptides, respectively. Parts of the datasets PXD018663, PXD019697 and PXD011284 were used for fine-tuning to further enhance the performance of the deep-learning model for MS/MS spectra prediction, as detailed in Supplementary Table 1. Non-phosphorylated peptides were included for fine-tuning, but not for test. Spectra used for fine-tuning and test had different embedding patterns.

To evaluate the performance of DeepFLR in FLR control, four synthetic phosphopeptides datasets were downloaded from PRIDE^50^ with the accession number PXD007058^72^, PXD000138^29^, PXD014525^37^ and PXD013210^73^ (Supplementary Table 2). For Syn_1 (PXD007058), the synthetic phosphopeptides were separated into five pools, and therefore only phosphopeptides belonging to their own pool were considered as correct identification. The same strategy was applied to Syn_2 (PXD000138) and Syn_4 (PXD013210). The raw files in Syn_2 were separated into the dataset (file name 1.raw to 48.raw) for fine-tuning and the test dataset (file name 49.raw to 96.raw). Each of the 96 raw files has different seed peptides. Thus, the spectra for fine-tuning and test have different peptide sequences. Syn_1 contains 809 PSMs of 130 phosphopeptides. The fine-tunning dataset of Syn_2 contains 62,906 PSMs of 24,186 phosphopeptides and 25,373 non-phosphopeptides, and the test dataset of Syn_2 contains 15,207 PSMs of 6537 phosphopeptides. Syn_3 contains 2546 PSMs of 184 phosphopeptides including 7 biphosphopeptide, 1 triphosphopeptide, and 1 tetraphosphopeptide. Syn_4 was acquired from a SCIEX TripleTOF 5600+ mass spectrometer, containing 21,778 PSMs of 1063 phosphopeptides.

To evaluate the performance of DeepFLR in the analysis of biological samples, three biological datasets were obtained (Supplementary Table 3). One biological dataset Bio_1 was generated by ourselves aforementioned. Two datasets were downloaded from PRIDE^50^ with the accession number PXD003344^35^ and PXD014525^37^. Bio_2 (PXD003344) was from MCF7 breast cancer cells. Bio_3 (PXD014525) was from EGF-stimulated REP1 cells treated with different MEK inhibitors. Part of the data from the biological datasets were used for fine-tuning the deep learning model. For Bio_1, 27,599 PSMs of 21,319 phosphopeptides and 3188 non-phosphopeptides were used for fine-tuning. For Bio_2, 11,387 PSMs of 8335 phosphopeptides and 1065 non-phosphopeptides were used for fine-tuning. For Bio_3, 34,278 PSMs of 18,106 phosphopeptides and 10,366 non-phosphopeptides were used for fine-tuning. It should be noted that the PSMs used for fine-tuning from the biological datasets may also be subjected to DeepFLR analysis.

To evaluate the performance of DeepFLR in DIA analysis, two DIA datasets were downloaded from PRIDE^50^ with the accession number PXD014525^37^ and from MassIVE proteomics repository (https://massive.ucsd.edu/) with project identifier MSV000082956^43^ (Supplementary Table 4). DIA_1 is a synthetic phosphopeptides DIA dataset acquired by a Q Exactive HF-X mass spectrometer, and its corresponding DDA dataset is Syn_3. DIA_2 is a biological sample dataset. DIA_2 and its corresponding DDA dataset were acquired by a Q Exactive HF mass spectrometer, with four DDA and DIA technical replicates, respectively.

### Protein sequence database searching

DDA data analysis was performed with different software solutions. SpectroMine (version 2.5.201125, Biognosys AG, Schlieren, Switzerland) analysis was performed with the following settings: Phospho (STY), Oxidation (M) and Acetyl (Protein N-term) as variable modifications; Carbamidomethyl (C) as fixed modification; PTM Localization Filter enabled. The others were default. Andromeda integrated in MaxQuant^8^ (version 1.6.17.0) was performed with the following settings: Phospho (STY), Oxidation (M) and Acetyl (Protein N-term) as variable modifications; Carbamidomethyl (C) as fixed modification. The others were default. The parameters for PEAKS (PEAKS studio version X + , Bioinformatics Solutions Inc., Waterloo, Canada) were: Phospho (STY), Oxidation (M) and Acetyl (Protein N-term) as variable modifications; Carbamidomethyl (C) as fixed modification; mass tolerance for the precursors as 20 ppm; maximum allowed variable PTM per peptide as 4; peptide level P-values <0.01. The other parameters were set as default. AscorePro was downloaded from https://github.com/gygilab/MPToolkit. The parameters for AscorePro were: Phospho (STY), Oxidation (M), and Acetyl (Protein N-term) as variable modifications; Carbamidomethyl (C) as fixed modification. The others were default. For phosphoRS (version 3.1), we conducted it using a command line interface phosphoRS-cli (https://github.com/lmsac/phosphoRS-cli). Carbamidomethyl (C) was set as a fixed modification, while Phospho (STY), Oxidation (M), and Acetyl (Protein N-term) were set as variable modifications. The other parameters were set default. LuciPHOr2 (JAVA Version 8 Update 321) was downloaded from http://luciphor2.sourceforge.net/. The parameters for LuciPHOr2 were: Phospho (STY), Oxidation (M), and Acetyl (Protein N-term) as variable modifications; Carbamidomethyl (C) as fixed modification; algorithm as 1, referring to the HCD-based method; minimum m/z for fragments as 0; maximum charge state as 3; neutral loss of phosphorylation considered; minimum score a PSM to be considered for modeling as 0.99. The others were default.

For PRM data analysis, raw files were analyzed by SpectroDive (version 11, Biognosys AG, Schlieren, Switzerland) with the default settings. SpectroDive calculated the ideal mass tolerances for data extraction and scoring based on its extensive mass calibration. Q-value cutoff on precursor was applied as 1%.

DIA datasets (DIA_1 and DIA_2) were searched by Spectronaut Enterprise x64^74^ (version 16.1.220730.53000, Biognosys AG, Schlieren, Switzerland) with PTM localization filter enabled. For spectral library construction, DDA datasets were searched by SpectroMine (version 2.5.201125, Biognosys AG, Schlieren, Switzerland). All the settings were default.

FASTA files were from UniProt *H. sapiens* reference proteome (access date 2019-12, 20,600 entries), UniProt *M. musculus* reference proteome (access date 2020-7, 17,082 entries), UniProt *A. thaliana* reference proteome (access date 2017-10, 27,468 entries), UniProt *S. cerevisiae* reference proteome (access date 2014-7, 108,460 entries) and UniProt *E. coli* reference proteome (access date 2020-7, 11,179 entries), respectively. For the synthetic peptide datasets, FASTA files were from the synthetic phosphopeptides sequences published by the corresponding papers.

### Deep learning model construction

The model of DeepFLR consists of four components: input, the embedding layer, BERT encoder and the output layer. Input is formed as (X, Y), where X is the sequence matrix of input phosphopeptides and Y is the corresponding MS/MS spectra. The final embedding is the sum of four embeddings of amino acids, charge, PTMs position and PTMs type. To simulate the fragmentation between amino acids, a special token “[SEP]” is used between every neighbor token. “[CLS]” is placed before every sequence. For peptides, “1” is used to represent phosphorylation (STY), “2” to represent oxidation (M), “3” to represent carbamidomethyl (C) and “4” to represent acetyl (protein N-terminal). The parameters for the BERT^26^ base model was downloaded from Hugging Face (https://huggingface.co/bert-base-uncased). The BERT encoder, it consists of twelve Transformer layers, each with two sublayers. The first sublayer is a multiheaded self-attention layer, and the second is a fully connected point-by-point feed-forward network. Residual connection and normalization are used in each sublayer. After the BERT encoder, an output layer is used to transform the hidden states into predicted MS/MS fragments. For each sequence, we extract hidden states from the token “[SEP]” to get the representation of every fragmentation position. Finally, we use a simple multi-layer perceptron (MLP) head to transform hidden states into the final predicted fragments. Mean square error (MSE) loss was used to optimize the model. Model construction was performed using python (3.8.3) with the following packages: FastNLP (0.6.0), pytorch (1.8.1), bidict (0.22.0), pyteomics (4.5.5), and transformers (4.12.5).

DeepFLR considers 36 types of fragment peaks (b1, bn1, bo1, b2, bn2, bo2, y1, yn1, yo1, y2, yn2, yo2, bp1, bnp1, bop1, bp2, bnp2, bop2, yp1, ynp1, yop1, yp2, ynp2, yop2, b2p1, bn2p1, bo2p1, b2p2, bn2p2, bo2p2, y2p1, yn2p1, yo2p1, y2p2, yn2p2, yo2p2). The first character means ion type (b ions or y ions), followed by neutral loss type (o means loss of H_2_O, n means loss of NH_3_, p means loss of H_3_PO_4_, op means loss of H_2_O and H_3_PO_4_, np means loss of NH_3_ and H_3_PO_4_, 2p means loss of two H_3_PO_4_, o2p means loss of one H_2_O and two H_3_PO_4_, n2p means loss of one NH_3_ and two H_3_PO_4_). The final character means fragment charge (1 or 2).

To train/retrain/fine-tune DeepFLR, DDA data were analyzed by SpectroMine. The intensity values were normalized to a 0-1 range and were log-transformed with log_2_ (x + 1), where x is the normalized intensity value. Training of DeepFLR using the dataset Train_1 took one day, and re-training of DeepFLR to fit Q-TOF using the dataset Train_2 took two hours on a single GPU RTX 3090 with batch size 128 and learning rate 2 × 10^−5^. Fine-tuning was processed for less than ten epochs with batch size 128 and learning rate 2 × 10^−5^.

### MS/MS prediction evaluation

Cosine similarity was calculated between the predicted and experimental spectra. The experimental spectra were converted to Mascot generic format (MGF) using MsConvert^75^ from the ProteoWizard Package (3.0.11579). Predicted spectra were generated by the deep-learning model. A tolerance of 25 ppm was set to recognize the common peaks between the experimental and the predicted spectra. If there was a discrepancy between two compared spectra, then the intensity for the missing peak was set as 0. The cosine similarity between the replicated experimental spectra for the same peptide was also calculated in the same way.

The published tools, i.e. pDeep2^22^ and DeepPhospho^23^, were also used for MS/MS spectra prediction. For pDeep2, the source code and the pre-trained model parameter pretrain-180921-modloss-transfer-Phos.ckpt were downloaded from the Github repository (https://github.com/pFindStudio/pDeep/tree/master/pDeep2), and the MS/MS spectra prediction was carried out according to the corresponding instruction. DeepPhospho was downloaded from the Github repository (https://github.com/weizhenFrank/DeepPhospho). RPE1_DDA provided in DeepPhospho was used as the model for MS/MS prediction without fine-tuning and PretrainParams was used for fine-tuning. The fine-tuning datasets of Test_1, Test_2 and Test_3 for DeepPhospho were the same as the ones used by DeepFLR (see details in Supplementary Table 1) and were transformed into the format of SNLib to fit the DeepPhospho. DeepPhospho was also fine-tuned using the same training dataset Train_1 used by DeepFLR using the RPE1_DDA model and configuration provided by DeepPhospho (https://github.com/weizhenFrank/DeepPhospho/blob/main/demo/ConfigDemo-IonModel-ModelTestWith_U2OS_DIA-Train.json). DeepPhospho filtered the predicted spectra of peptides with few fragments. Here, we treated the missing value of DeepPhospho as zero.

### Identification of phosphopeptides by DeepFLR

After database searching, the identified MS/MS of phosphopeptides (localization probability score threshold = 0, with No. of candidate phosphosites > No. of phosphate groups) were extracted for DeepFLR analysis. The target phosphopeptides were generated by placing phosphate groups on all candidate residues to generate all possible isoforms of the identified phosphopeptides by database searching. For each target phosphopeptide, the corresponding decoy phosphopeptide was generated by exchanging the phosphorylated amino acid residue with another amino acid residue randomly. Cosine similarity was calculated between each experimental spectrum and the predicted spectra of its corresponding candidate target and decoy phosphopeptides. The identification result was reported as the target or decoy phosphopeptide with the highest cosine similarity score, and the delta score was calculated as the difference value between the maximum cosine similarity and the closest cosine similarity of a target phosphopeptide. Identification results of all experimental spectra were ranked by the delta score, and the FLR was calculated as the ratio of decoys among all the identification results above a certain delta score threshold adjusted by the numbers of all candidate decoys and target phosphopeptides:

1 $$\begin{array}{c}{{{{{{\rm{FLR}}}}}}}_{{{{{{\rm{estimated}}}}}}}=\frac{{N}_{{{{{{\rm{decoy}}}}}}}+{N}_{{{{{{\rm{target}}}}}}}}{{N}_{{{{{{\rm{decoy}}}}}}}}\times \frac{{{{{{\rm{\#D}}}}}}}{{{{{{\rm{\#T}}}}}}+{{{{{\rm{\#D}}}}}}}\end{array}$$

where *N*_decoy_ is the total number of decoy in the database; *N*_*t*arget_ is the total number of targets in the database; #D is the number of identified decoy hits; and #T is the number of identified target hits. To evaluate the performance of the FLR estimation, synthetic phosphopeptides datasets were used to calculate the real FLR:

2 $$\begin{array}{c}{{{{{{\rm{FLR}}}}}}}_{{{{{{\rm{syn}}}}}}}=\frac{{{{{{\rm{False\; Positive}}}}}}}{{{{{{\rm{True\; Positive}}}}}}+{{{{{\rm{False\; Positive}}}}}}}\end{array}$$

where False Positive refers to the count of PSMs not matching the synthetic phosphopeptide sequence and True Positive refers to the count of PSMs matching the synthetic phosphopeptide sequence. The synthetic phosphopeptides datasets were repeatedly analyzed by DeepFLR (*n* = 10) to assess the randomicity of DeepFLR in FLR estimation. The 95% confidence interval was calculated:

3 $$\begin{array}{c}\mu=\bar{x}\pm 2.26\sqrt{\frac{\sum {\left({x}_{i}-\bar{x}\right)}^{2}}{n\left(n-1\right)}}\end{array}$$

where 2.26 is the coefficient α based on the degree of freedom (*f* = 9) for bilateral experiments; *n* is the observed number of PSMs; *x* is the measured value. If only one experiment had a valid value at a specific cutoff, the confidence interval was set as 0. Data analysis in this part was performed using python (3.8.3) with the following packages: pandas (1.0.5) and numpy (1.18.5).

### Construction of spectral libraries for DIA analysis

For spectral library construction for DIA analysis, corresponding DDA datasets were searched by SpectroMine (version 2.5.201125, Biognosys AG, Schlieren, Switzerland) with a localization probability threshold of 0, and the results were submitted to DeepFLR for re-localization with an estimated FLR of 0.01. For a predicted spectral library, all MS/MS spectra were predicted by DeepFLR. For the hybrid spectral library, if DeepFLR identified a different phosphopeptide compared to SpectroMine from an MS/MS spectrum, the MS/MS spectrum of the phosphopeptide in the library was generated by DeepFLR prediction, while the others were from the experiment. For the re-localized experimental spectral library, all MS/MS spectra were from the experiment but the phosphopeptide identification result was from DeepFLR. All the retention time values for the three libraries were from the experiment. For the SpectroMine-based experimental spectral library, the DDA results were filtered with a localization probability of default value (0.75). DIA data were then analyzed by Spectronaut Enterprise x64^74^ (version 16.1.220730.53000, Biognosys AG, Schlieren, Switzerland) with PTM localization filter enabled using the DeepFLR-based spectral library or the SpectroMine-based experimental spectral library.

For comparison, DeepPhospho was also used to generate a predicted spectral library. The phosphopeptides in the library were obtained by SpectroMine with 0.75 localization probability threshold. Both the MS/MS spectra and the retention time values were predicted by DeepPhospho using RPE1_DDA model.

### Statistics and bioinformatics analysis

For the dataset Bio_2, kinase-reacted sites were identified by matching the localized phosphosites to the kinase database. Kinase information (Kinase_Substrate_Dataset.txt) was downloaded from PhosphoSitePlus^76^ (access date 2021-12). The sequence logo was constructed by WebLogo (version 3.7.4). The type was set as protein and units were set as probability. For the dataset Bio_3, DeepFLR and MaxQuant results were processed to produce a.txt file of localized protein phosphosites quantification matrix to be compatible with Perseus (version 1.6.15.0). The quantification information of each protein phosphosite was the summed quantification information of all phosphopeptides corresponding to the protein phosphosite. Processed by Perseus, only protein phosphosites with a minimum of two valid values in at least one treatment group (no treatment, EGF treated only, and EGF treated with different MEK inhibitors of Cobimetinib (5 μM or 0.5 μM) or PD0325901 (5 μM or 0.5 μM)) were kept, and then subjected to log2(x) transformation and z-score normalization. Missing values were imputed by randomly sampling the lower end of the normal distribution (width 0.3 and downshift 1.8 as the default settings of Perseus) in the whole matrix. One-way ANOVA significance test was performed with parameter settings of s0 = 0 and Permutation-based FDR = 0.05 to identify significantly regulated sites with ANOVA q-value <0.05. Heatmaps of all significantly regulated phosphorylation sites were generated by unsupervised hierarchical clustering using Euclidean distance. The sequence logo was constructed by WebLogo (version 3.7.4). The type was set as protein and units were set as bits. The kinase substrate information used to draw the sequence logo of ERK1/2 substrates was from the aforementioned kinase database (Kinase_Substrate_Dataset.txt). Visualization was performed using custom scripts in R (4.0.2) with the following packages: VennDiagram (1.6.20), ComplexHeatmap (2.13.2) and ggplot2 (3.3.2). For coefficient of variation (CV) calculation in DIA analysis, the quantification was denormalized and then uploaded to Perseus to calculate phosphosite stoichiometry by a custom-coded plugin Peptide collapse (version 1.4.4) downloaded from https://github.com/AlexHgO/Perseus_Plugin_Peptide_Collapse.

### Reporting summary

Further information on research design is available in the Nature Portfolio Reporting Summary linked to this article.

## Supplementary information

Supplementary information

Description of Additional Supplementary Files

Supplementary Dataset 1

Supplementary Dataset 2

Supplementary Dataset 3

Supplementary Dataset 4

Supplementary Dataset 5

Supplementary Dataset 6

Supplementary Dataset 7

Reporting Summary

## Data Availability

All the LC-MS/MS raw data, FASTA files, search results, saved projects and database searching parameters generated in this study have been deposited to ProteomeXchange via the iProX^77^ partner repository under accession code PXD037580 or IPX0005248000. The 14 raw datasets used in this study for deep learning model pre-training are available in the PRIDE^50^ database under accession code PXD004452^51^, PXD001374^52^, PXD001305^53^, PXD003529^54^, PXD002135^55^, PXD004447^56^, PXD000612^57^, PXD001565^58^, PXD004252^59^, PXD001550^60^, PXD001546^60^, PXD002286^61^, PXD003531^62^ and PXD002394^63^. The three datasets used in this study for model re-training to fit the Q-TOF data are available in the PRIDE^50^ database under accession code PXD006056^64^, PXD012433^65^, and PXD015687^66^. The five datasets used in this study for evaluating the performance of DeepFLR in MS/MS spectra prediction are available in the PRIDE^50^ database with the accession number PXD018663 (Test_1)^67^, PXD019697 (Test_2)^68^, PXD011284 (Test_3)^69^, PXD023361 (Test_4)^70^ and PXD008211 (Test_5)^71^. The four synthetic phosphopeptides datasets used in this study to evaluate the performance of DeepFLR in FLR control are available in the PRIDE^50^ database with the accession number PXD007058 (Syn_1)^72^, PXD000138 (Syn_2)^29^, PXD014525 (Syn_3)^37^ and PXD013210 (Syn_4)^73^. Two external biological datasets used in this study to evaluate the performance of DeepFLR in the analysis of biological samples are available in the PRIDE^50^ database with the accession number PXD003344 (Bio_2)^35^ and PXD014525 (Bio_3)^37^. Two DIA datasets used in this study to evaluate the performance of DeepFLR in DIA analysis are available in the PRIDE^50^ database with the accession number PXD014525 (DIA_1)^37^ and from MassIVE proteomics repository with project identifier MSV000082956 [https://massive.ucsd.edu/ProteoSAFe/dataset.jsp?task=92965d2a3515472aa16100fa2525928d] (DIA_2)^43^. The source data underlying all figures except for those not including statistics are provided as a Source Data file. Source data are provided with this paper.

## Source data

Source data

## Code availability

DeepFLR is open source and freely available on GitHub [https://github.com/lmsac/DeepFLR] and Zenodo [10.5281/zenodo.7777409]^78^.
