# Supplementary material for: DeepFLR facilitates false localization rate control in phosphoproteomics
Source: Nat Commun. 2023 Apr 20;14:2269. doi: 10.1038/s41467-023-38035-1 (PMC10119288; doi:10.1038/s41467-023-38035-1)
Supplement: Supplementary file 1 — Supplementary information [file 41467_2023_38035_MOESM1_ESM.pdf]

Supplementary Information for

DeepFLR Facilitates False Localization Rate Control in Phosphoproteomics

Zong et al.

Supplementary Table 1. Datasets for deep learning model training and evaluation.

Name	Species	Instrument	Accession	Description
Train_1	Human	Q Exactive/ Q Exactive HF/ Q Exactive Plus/ Orbitrap Fusion/	PXD004452[1]<br>PXD001374[2]<br>PXD001305[3]<br>PXD003529[4]<br>PXD002135[5]<br>PXD004447[6]<br>PXD000612[7]<br>PXD001565[8]<br>PXD004252[9]<br>PXD001550[10]<br>PXD001546[10]<br>PXD002286[11]<br>PXD003531[12]<br>PXD002394[13]	Training dataset:<br>Mono-phosphopeptides: 163,543 PSMs, 120,450 phosphopeptides<br>Multi-phosphopeptides: 82,902 PSMs, 63,580 phosphopeptides<br>Non-phosphopeptides: 221,139 PSMs, 165,786 peptides<br>Total: 467,584 PSMs, 184,030 phosphopeptides<br><br>Validation dataset:<br>Mono-phosphopeptides: 18,090 PSMs, 17,491 phosphopeptides<br>Multi-phosphopeptides: 9360 PSMs, 9090 phosphopeptides<br>Non-phosphopeptides: 24,519 PSMs, 23,750 peptides<br>Total: 51,969 PSMs, 26,581 phosphopeptides
Test_1	Human	Q Exactive	PXD018663[14]	Mono-phosphopeptides: 13,891 PSMs, 11,317 phosphopeptides<br>Multi-phosphopeptides: 1955 PSMs, 1651 phosphopeptides<br>Non-phosphopeptides: 10,803 PSMs, 8969 peptides<br>Total: 26,649 PSMs, 12,968 phosphopeptides<br><br>Fine-tune (Training dataset/ Test dataset):<br>Training dataset:<br>Mono-phosphopeptides: 6833 PSMs, 6595 phosphopeptides<br>Multi-phosphopeptides: 750 PSMs, 732 phosphopeptides<br>Non-phosphopeptides: 5741 PSMs, 5622 peptides<br>Total: 13,324 PSMs, 7327 phosphopeptides<br><br>Test dataset:<br>Mono-phosphopeptides: 7058 PSMs, 6510 phosphopeptides<br>Multi-phosphopeptides: 1205 PSMs, 1135 phosphopeptides<br>Total: 8263 PSMs, 7645 phosphopeptides
Test_2	Mouse	Q Exactive HF	PXD019697[15]	Mono-phosphopeptides: 12,707 PSMs, 10,704 phosphopeptides<br>Multi-phosphopeptides: 13,907 PSMs, 11,733 phosphopeptides<br>Non-phosphopeptides: 1374 PSMs, 1220 peptides

				Total: 27,988 PSMs, 22,437 phosphopeptides<br><br>Fine-tune (Training dataset/Test dataset):<br>Training dataset:<br>Mono-phosphopeptides: 7351 PSMs, 6828 phosphopeptides<br>Multi-phosphopeptides: 5728 PSMs, 5291 phosphopeptides<br>Non-phosphopeptides: 915 PSMs, 864 peptides<br>Total: 13,994 PSMs, 12,119 phosphopeptides<br><br>Test dataset:<br>Mono-phosphopeptides: 5356 PSMs, 4938 phosphopeptides<br>Multi-phosphopeptides: 8179 PSMs, 7524 phosphopeptides<br>Total: 13,535 PSMs, 12,462 phosphopeptides
Test_3	Mouse-ear cress	Q Exactive	PXD011284[16]	Mono-phosphopeptides: 2399 PSMs, 2171 phosphopeptides<br>Multi-phosphopeptides: 576 PSMs, 503 phosphopeptides<br>Non-phosphopeptides: 31,676 PSMs, 27,665 peptides<br>Total: 34,651 PSMs, 2674 phosphopeptides<br><br>Fine-tune (Training dataset/ Testing dataset):<br>Training dataset:<br>Mono-phosphopeptides: 788 PSMs, 785 phosphopeptides<br>Multi-phosphopeptides: 115 PSMs, 113 phosphopeptides<br>Non-phosphopeptides: 16,422 PSMs, 16,397 peptides<br>Total: 17,325 PSMs, 898 phosphopeptides<br><br>Test dataset:<br>Mono-phosphopeptides: 1611 PSMs, 1514 phosphopeptides<br>Multi-phosphopeptides: 461 PSMs, 421 phosphopeptides<br>Total: 2072 PSMs, 1935 phosphopeptides
Test_4	Yeast	Q Exactive HF	PXD023361[17]	Mono-phosphopeptides: 162 PSMs, 132 phosphopeptides<br>Multi-phosphopeptides: 5 PSMs, 4 phosphopeptides<br>Non-phosphopeptides: 2991 PSMs, 2000 peptides<br>Total: 3158 PSMs, 136 phosphopeptides

Test_5	E. coli	Q Exactive HF	PXD008211[18]	Mono-phosphopeptides: 73 PSMs, 64 phosphopeptides<br>Multi-phosphopeptides: 5 PSMs, 5 phosphopeptides<br>Non-phosphopeptides: 2404 PSMs, 2147 peptides<br>Total: 2482 PSMs, 69 phosphopeptides

The numbers of PSMs and phosphopeptides are based on the database searching software solution and filtration conditions detailed in Methods section.

Supplementary Table 2. The datasets of synthetic phosphopeptides used for performance comparison and the datasets used for re-training of DeepFLR to fit Q-TOF.

Name	Instrument	Accession	Description
Syn_1	Orbitrap Fusion	PXD007058[19]	HCD MS/MS spectra of synthetic monophosphopeptides<br>pS: 500 PSMs, 84 phosphopeptides<br>pT: 123 PSMs, 17 phosphopeptides<br>pY: 186 PSMs, 29 phosphopeptides<br>Total: 809 PSMs, 130 phosphopeptides, 96 sequences<br>Average candidate sites per phosphopeptide: 3.5
Syn_2	Orbitrap LTQ Velos	PXD000138[20]	HCD MS/MS spectra of synthetic monophosphopeptides<br>Data for fine-tunning:<br>The raw files with file name 1.raw to 48.raw.<br><br>Test dataset:<br>The raw files with file name 49.raw to 96.raw.<br>pS: 4256 PSMs, 1980 phosphopeptides<br>pT: 3722 PSMs, 1795 phosphopeptides<br>pY: 7229 PSMs, 2762 phosphopeptides<br>Total: 15,207 PSMs, 6537 phosphopeptides, 6537 sequences<br>Average candidate sites per phosphopeptide: 3.0
Syn_3	Q Exactive HF-X	PXD014525[21]	HCD MS/MS spectra of synthetic monophosphopeptides and multiphosphopeptides<br>Total: 2546 PSMs, 175 monophosphopeptides, 7 biphosphopeptide, 1 triphosphopeptide, 1 tetraphosphopeptide<br>Average candidate sites per monophosphopeptide: 3.1<br>Average candidate sites per biphosphopeptide: 4.9<br>Average candidate sites per triphosphopeptide: 4<br>Average candidate sites per tetraphosphopeptide: 6
Syn_4	TripleTOF 5600+	PXD013210[22]	HCD MS/MS spectra of synthetic monophosphopeptides<br>pS: 10,794 PSMs, 555 phosphopeptides<br>pT: 6092 PSMs, 275 phosphopeptides<br>pY: 4892 PSMs, 233 phosphopeptides<br>Total: 21,778 PSMs, 1063 phosphopeptides, 361 sequences<br>Average candidate sites per phosphopeptide: 4.2

Train_2	TripleTOF 6600	PXD006056[23]<br>PXD012433[24]<br>PXD015687[25]	Training dataset:<br>Mono-phosphopeptides: 17,051 PSMs, 14,033 phosphopeptides<br>Multi-phosphopeptides: 2754 PSMs, 2264 phosphopeptides<br>Non-phosphopeptides: 14,272 PSMs, 12,471 peptides<br>Total: 34,077 PSMs, 16,297 phosphopeptides<br><br>Validation dataset:<br>Mono-phosphopeptides: 1916 PSMs, 1872 phosphopeptides<br>Multi-phosphopeptides: 299 PSMs, 294 phosphopeptides<br>Non-phosphopeptides: 1571 PSMs, 1550 peptides<br>Total: 3786 PSMs, 2166 phosphopeptides

The numbers of PSMs and phosphopeptides are based on the database searching software solution and filtration conditions detailed in Methods section.

Supplementary Table 3. The three biological datasets.

Name	Instrument	Accession	Description
Bio_1	Orbitrap Fusion Lumos Tribrid	PXD037580	HCD MS/MS spectra of Hela cells<br><br>Data for fine-tunning:<br>Mono-phosphopeptides: 15,770 PSMs, 13,987 phosphopeptides<br>Multi-phosphopeptides: 8281 PSMs, 7332 phosphopeptides<br>Non-phosphopeptides: 3548 PSMs, 3188 peptides<br>Total: 27,599 PSMs, 21,319 phosphopeptides
Bio_2	Q Exactive	PXD003344[26]	HCD MS/MS spectra of MCF7 breast cancer cells<br><br>Data for fine-tunning:<br>Mono-phosphopeptides: 9979 PSMs, 8213 phosphopeptides<br>Multi-phosphopeptides: 142 PSMs, 122 phosphopeptides<br>Non-phosphopeptides: 1266 PSMs, 1065 peptides<br>Total: 11,387 PSMs, 8335 phosphopeptides
Bio_3	Q Exactive HF-X	PXD014525[21]	HCD MS/MS spectra of epidermal growth factor (EGF)-stimulated retinal pigment epithelium (RPE1) cells with six conditions of no treatment, EGF treated only, EGF treated with Cobimetinib (5 µM or 0.5 µM) or EGF treated with PD0325901 (5 µM or 0.5 µM).<br><br>Data for fine-tunning:<br>Mono-phosphopeptides: 15,795 PSMs, 12,661 phosphopeptides<br>Multi-phosphopeptides: 6807 PSMs, 5445 phosphopeptides<br>Non-phosphopeptides: 11,676 PSMs, 10,366 peptides<br>Total: 34,278 PSMs, 18,106 phosphopeptides

The numbers of PSMs and phosphopeptides are based on the database searching software solution and filtration conditions detailed in Methods section.

Supplementary Table 4. The DIA datasets used for evaluating the performance of DeepFLR-based spectral libraries for DIA analysis.

Name	Instrument	Accession	Description
DIA_1	Q Exactive HF-X	PXD014525[21]	Synthetic phophopeptides DIA datasets with Syn_3 (Supplementary Table 2) as its corresponding DDA dataset
DIA_2	Q Exactive HF	MSV000082956[27]	phophopeptides DIA datasets and corresponding DDA datasets of biological samples<br><br>Data for fine-tunning:<br>Total: 19,792 PSMs, 12,828 phosphopeptides, 3212 non-phosphorylated peptides

The numbers of PSMs and phosphopeptides are based on the database searching software solution and filtration conditions detailed in Methods section.

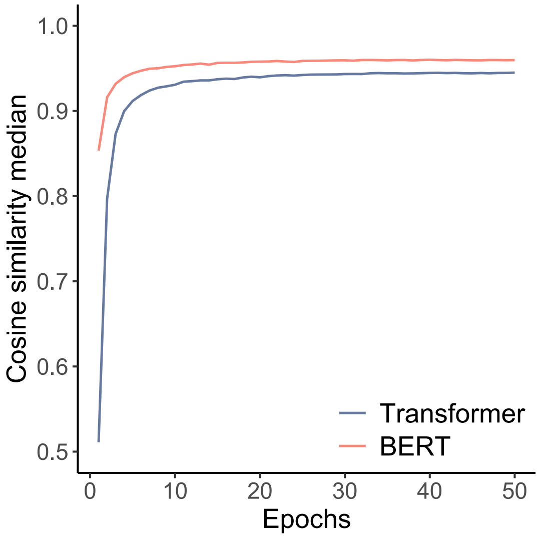

Supplementary Figure 1. Comparison of Transformer and BERT. Performance of Transformer or BERT during model training in MS/MS spectra prediction. The cosine similarity median refers to the median cosine similarity computed between the predicted and experimental spectra for each epoch on the validation dataset split from Train_1. The BERT model weights were downloaded from https://huggingface.co/bert-base-uncased using python package transformers (4.12.5). The Transformer model used the same structure but model weights were initialized by the built-in Xavier initialization method. Model training was conducted on a single GPU RTX 3090 with batch size 128 and learning rate 2×10^{-5}. Source data are provided as a Source Data file.

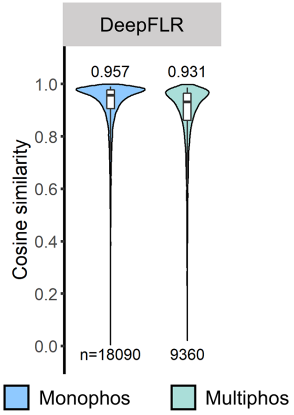

Supplementary Figure 2. Performance of DeepFLR in MS/MS spectra prediction on the validation dataset of Train_1. The distribution of cosine similarity was computed between the predicted and experimental spectra for monophosphopeptides and multiphosphopeptides, respectively, on the validation dataset split from Train_1. The medians are indicated. The boxes and whiskers show the quantiles and 95% percentiles, respectively. The numbers of spectra for test are indicated below each graph. Source data are provided as a Source Data file.

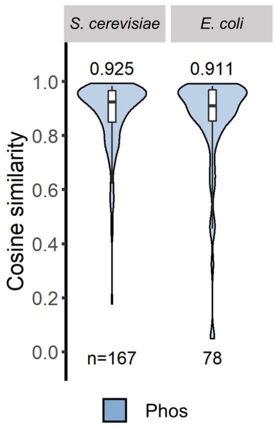

Supplementary Figure 3. Performance of DeepFLR in MS/MS prediction with the datasets from *Saccharomyces cerevisiae* and *Escherichia coli.* The distribution of cosine similarity was computed between the predicted and experimental spectra from the datasets of *S cerevisiae* (Test_4) and *E. coli* (Test_5), respectively. The medians are indicated. The boxes and whiskers show the quantiles and 95% percentiles, respectively. The numbers of spectra for test are indicated below each graph. Source data are provided as a Source Data file.

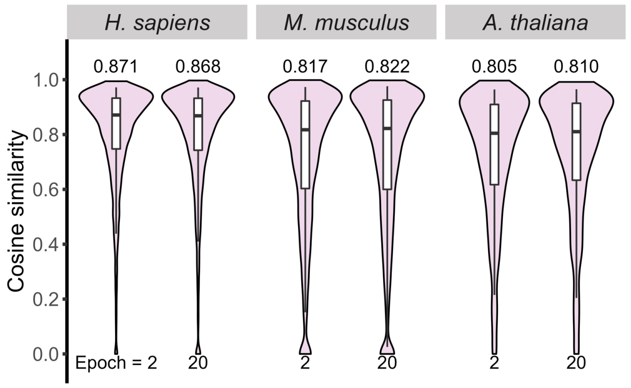

Supplementary Figure 4. Fine-tuning of DeepPhospho with epochs of 2 or 20 for MS/MS prediction. The distribution of cosine similarity was computed between the predicted and experimental spectra from the datasets of *H. sapiens* (Test_1), *M. musculus* (Test_2) and *A. thaliana* (Test_3), respectively. The prediction was performed by DeepPhospho after fine-tuning with the epochs of 2 or 20. For DeepPhospho Desktop, the use of 2 epochs is recommended for fast fine-tuning and the use of 20 epochs is the default setting. The medians are indicated. The boxes and whiskers show the quantiles and 95% percentiles, respectively. Missing values from DeepPhospho were inputted as 0. Source data are provided as a Source Data file.

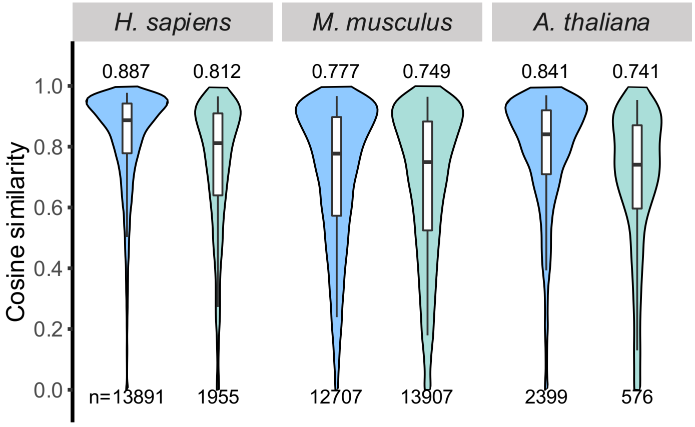

Supplementary Figure 5. Performance of DeepPhospho fine-tuned using Train_1. The distribution of cosine similarity was computed between the predicted and experimental spectra from the datasets of *H. sapiens* (Test_1), *M. musculus* (Test_2) and *A. thaliana* (Test_3), respectively. DeepPhospho was fine-tuned using the dataset Train_1. The model performed the best on the validation dataset of Train_1 was selected. The medians are indicated. The boxes and whiskers show the quantiles and 95% percentiles, respectively. The numbers of spectra used are indicated below each graph. Missing values from DeepPhospho were inputted as 0. Source data are provided as a Source Data file.

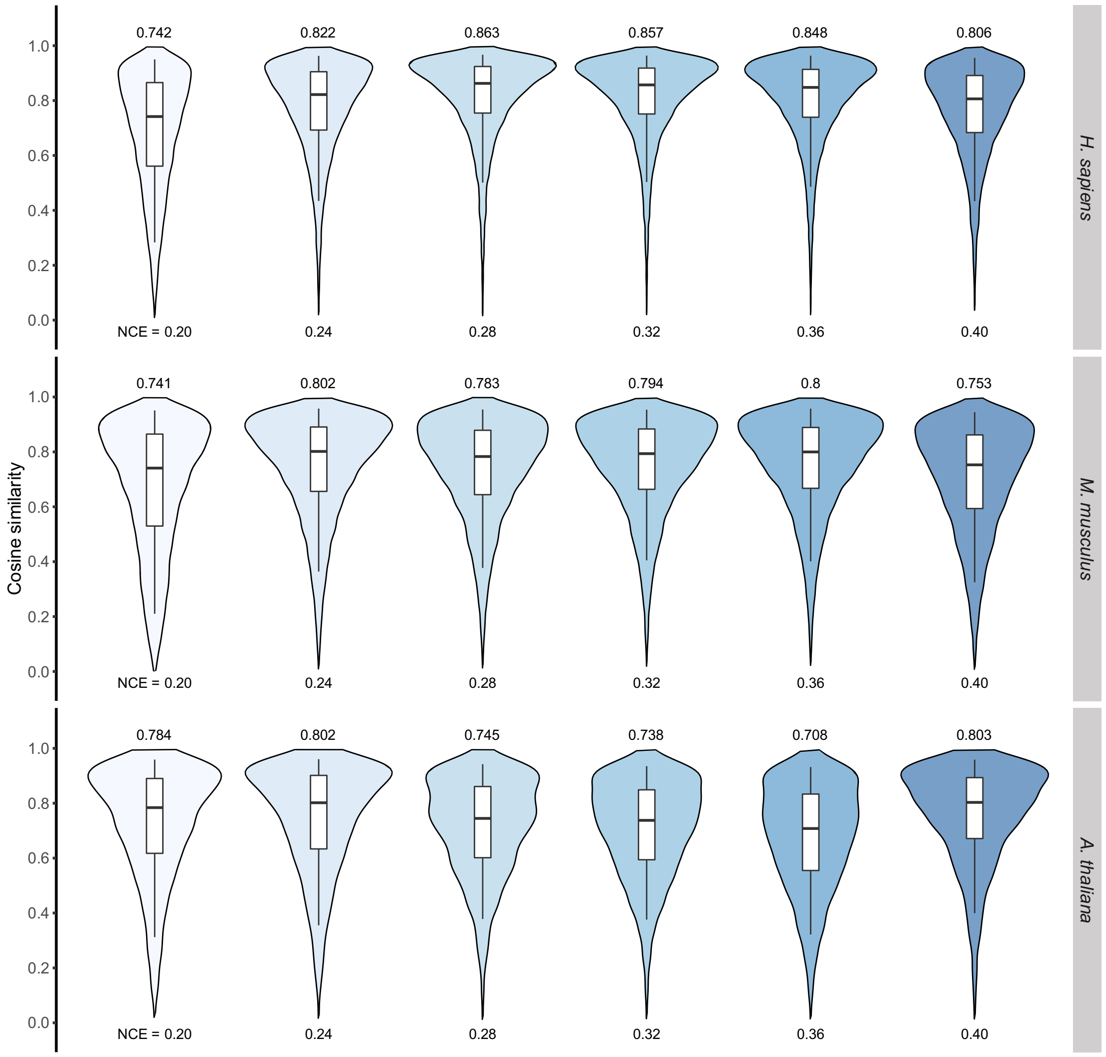

Supplementary Figure 6. Performance of pDeep2 with different normalized collisional energy (NCE) for MS/MS spectra prediction. The distribution of cosine similarity was computed between the predicted and experimental spectra from the datasets of *H. sapiens* (Test_1), *M. musculus* (Test_2) and *A. thaliana* (Test_3), respectively. The medians are indicated. The boxes and whiskers show the quantiles and 95% percentiles, respectively. The NCE used is indicated below each graph. Source data are provided as a Source Data file.

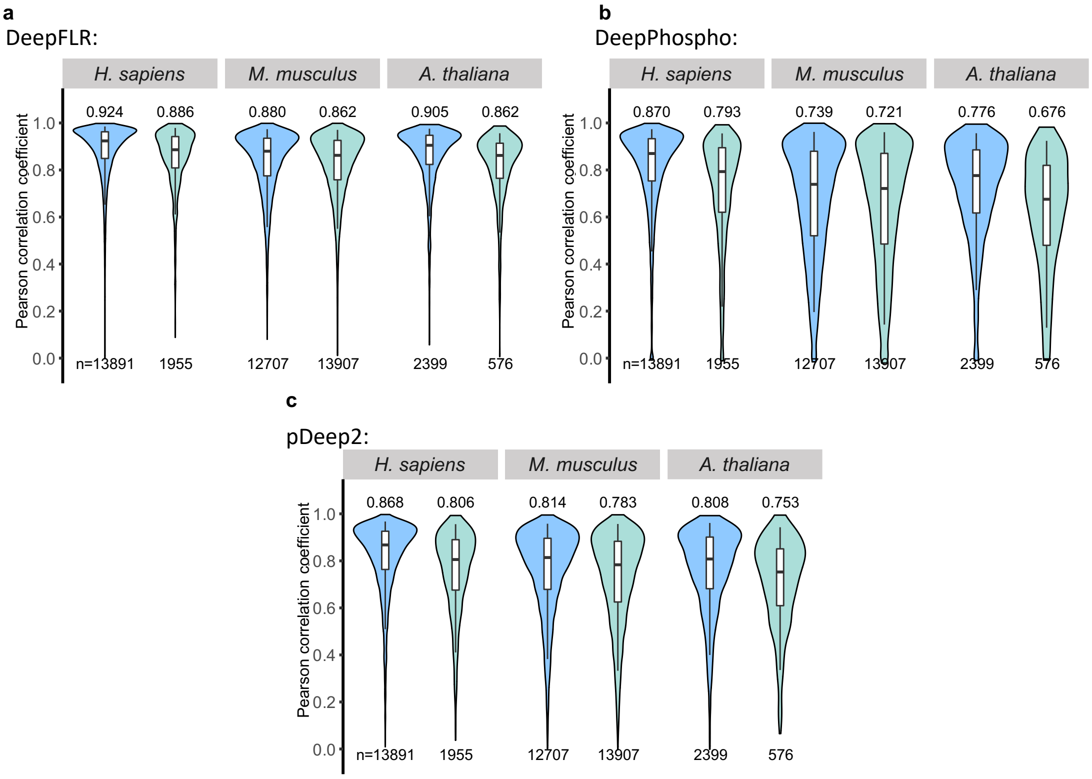

Supplementary Figure 7. The distribution of Pearson correlation coefficient computed between the predicted and experimental spectra from the datasets of *H. sapiens* (Test_1), *M. musculus* (Test_2) and *A. thaliana* (Test_3) for (a) DeepFLR, (b) DeepPhospho, and (c) pDeep2. The medians are indicated. The boxes and whiskers show the quantiles and 95% percentiles, respectively. The numbers of spectra used are indicated below each graph. The deep learning models of DeepFLR, DeepPhospho, and pDeep2 were same as those in **Figure 1** without any fine-tunning. Missing values from DeepPhospho were inputted as 0. For pDeep2, the normalized collision energy (NCE) parameters were set as 0.28, 0.24 and 0.40 for the *H. sapiens*, *M. musculus* and *A. thaliana* datasets, respectively. Source data are provided as a Source Data file.

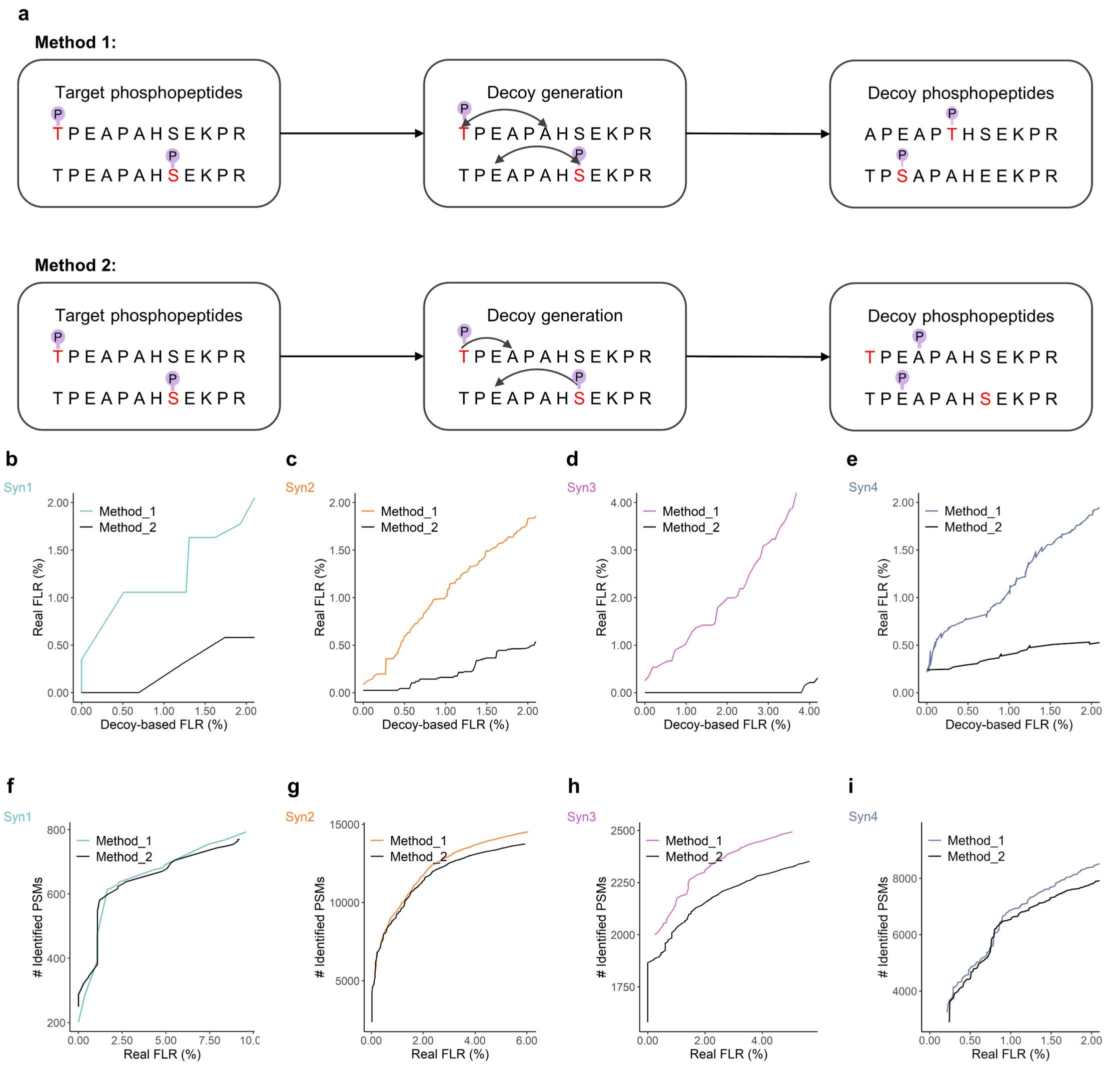

Supplementary Figure 8. Performance comparison of different decoy generation methods. **(a)** Depiction of the two different decoy generation methods. Method 1, exchanging the whole phosphorylated amino acid residue with another non-candidate amino acid residue in the target sequence; Method 2, randomly shifting the phosphate group to another non-candidate amino acid residue in the target sequence. **(b-e)** Estimated FLR by Method 1 and Method 2 plotted against the real FLR based on four synthetic phosphopeptides datasets. **(f-i)** Comparison of the number of identified phosphopeptides under a given real FLR by Method 1 and Method 2 on the four synthetic phosphopeptides datasets. Source data are provided as a Source Data file.

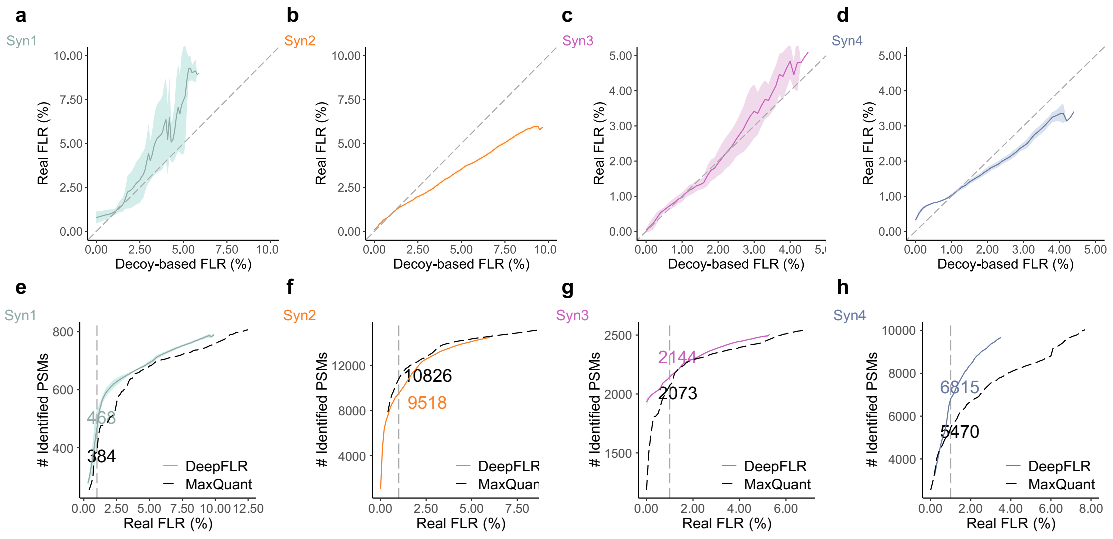

Supplementary Figure 9. (a-d) Estimated FLR plotted against the real FLR with 95% confidence interval in full range on four synthetic phosphopeptides datasets. The gray dashed line indicates the line where the estimated FLR is equal to the real FLR. **(e-h)** Comparison of the number of identified phosphopeptides PSMs under a given real FLR by DeepFLR with 95% confidence interval and by MaxQuant on the four synthetic phosphopeptides datasets. The numbers of identified PSMs at 1% real FLR are indicated. The colored solid line indicates the mean value of real FLR or identified PSMs, and the shaded area around the solid line indicates the 95% confidence interval of real FLR or identified PSMs. Source data are provided as a Source Data file.

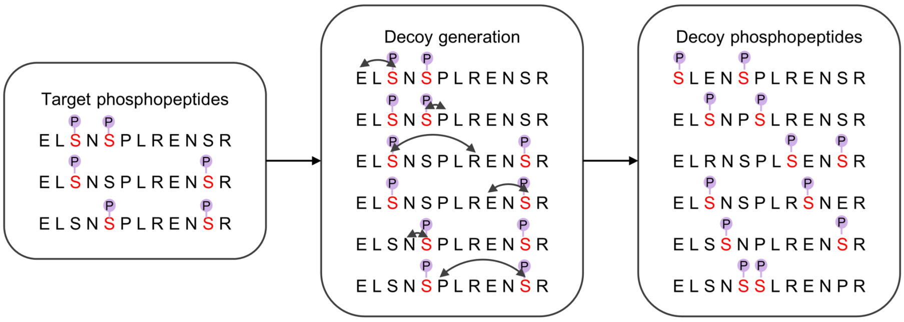

$$\mathrm{FLR_{estimated}} = \frac{N_{\mathrm{decoy}} + N_{\mathrm{target}}}{N_{\mathrm{decoy}}} \times \frac{\#\mathrm{D}}{\#\mathrm{T} + \#\mathrm{D}}$$

Supplementary Figure 10. The decoy generation and FLR estimation for multi-phosphorylated peptides. N_{decoy} is the total number of decoys in the database; N_{target} is the total number of targets in the database; #D is the number of identified decoy hits; #T is the number of identified target hits.

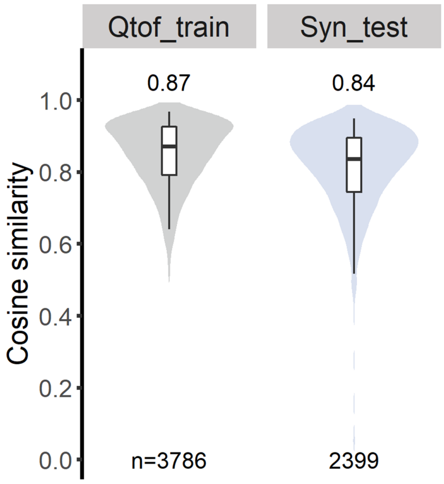

Supplementary Figure 11. Performance of DeepFLR in MS/MS prediction for Q-TOF datasets. Train_2 was divided into the training dataset and the validation dataset with a spectra ratio of about 9:1. The distribution of cosine similarity was computed between the predicted and experimental spectra from the validation dataset of Train_2 (Qtof_train) and Syn_4 (Syn_test), respectively. The medians are indicated. The boxes and whiskers show the quantiles and 95% percentiles, respectively. The numbers of spectra used are indicated below each graph. Source data are provided as a Source Data file.

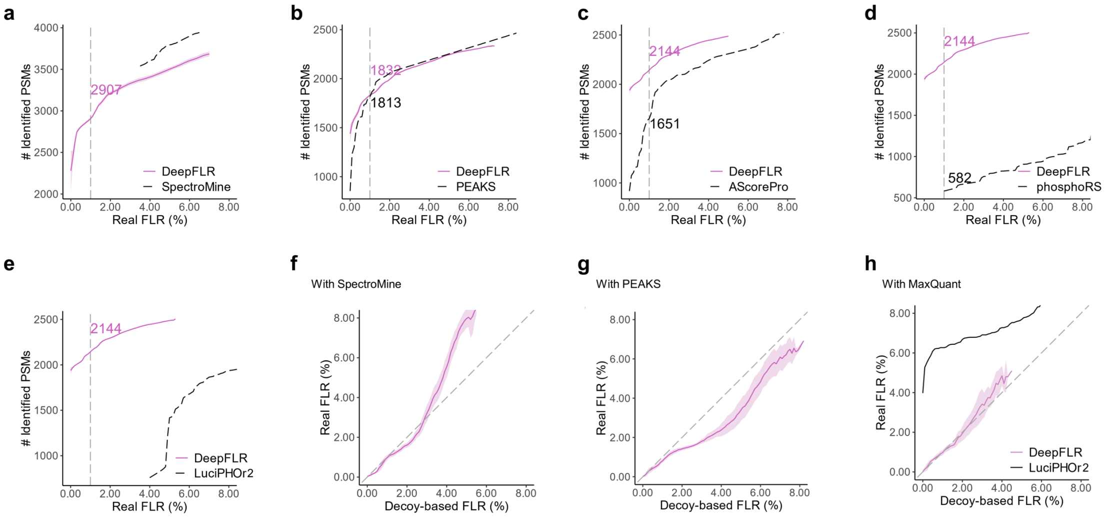

Supplementary Figure 12. Comparison of DeepFLR with SpectroMine, PEAKS, AscorePro, PhosphoRS, and LuciPHOr2 on Syn_3. The number of identified PSMs under a given real FLR by DeepFLR in combination with **(a)** SpectroMine or **(b)** PEAKS with 95% confidence interval or by SpectroMine or PEAKS alone. The number of identified PSMs under a given real FLR by DeepFLR in combination with MaxQuant with 95% confidence interval or by **(c)** AscorePro or **(d)** PhosphoRS. **(e)** The number of identified PSMs under a given real FLR by DeepFLR with 95% confidence interval or by LuciPHOr2 in combination with MaxQuant. Estimated FLR plotted against the real FLR with 95% confidence interval based on **(f)** DeepFLR in combination with SpectroMine, **(g)** DeepFLR in combination with PEAKS, and **(h)** DeepFLR or LuciPHOr2 in combination with MaxQuant. The numbers of identified PSMs at 1% real FLR are indicated. The gray dashed line indicates the line where the estimated FLR is equal to the real FLR. The colored solid line indicates the mean value of real FLR or identified PSMs, and the shaded area around the solid line indicates the 95% confidence interval of real FLR or identified PSMs. Source data are provided as a Source Data file.

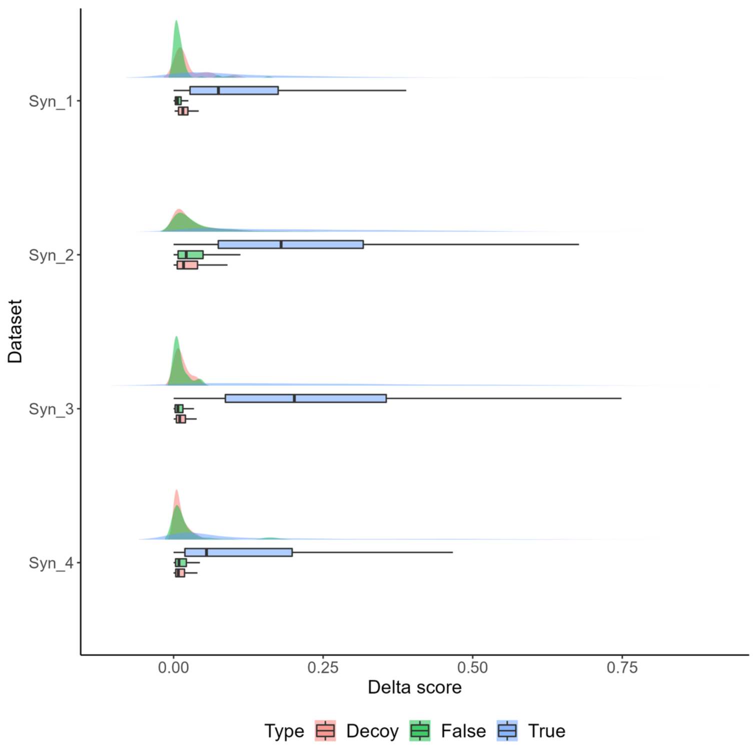

Supplementary Figure 13. Delta score distribution of decoy, false and true phosphopeptides hits. Half violin gram (Top) and boxplot (Bottom) of delta score distribution for decoy, true and false phosphopeptides hits. Boxes mark the first and third quartile, with the median highlighted as the line, and whiskers mark the minimum/maximum values within the 1.5 interquartile range. Outliers are not shown. For Syn_1, n=717, 76 and 16 respectively for True, False and Decoy hits. For Syn_2, n=13628, 882 and 697 respectively for True, False and Decoy hits. For Syn_3, n=2367, 126 and 53 respectively for True, False and Decoy hits. For Syn_4, n=9556, 103 and 392 respectively for True, False and Decoy hits. Source data are provided as a Source Data file.

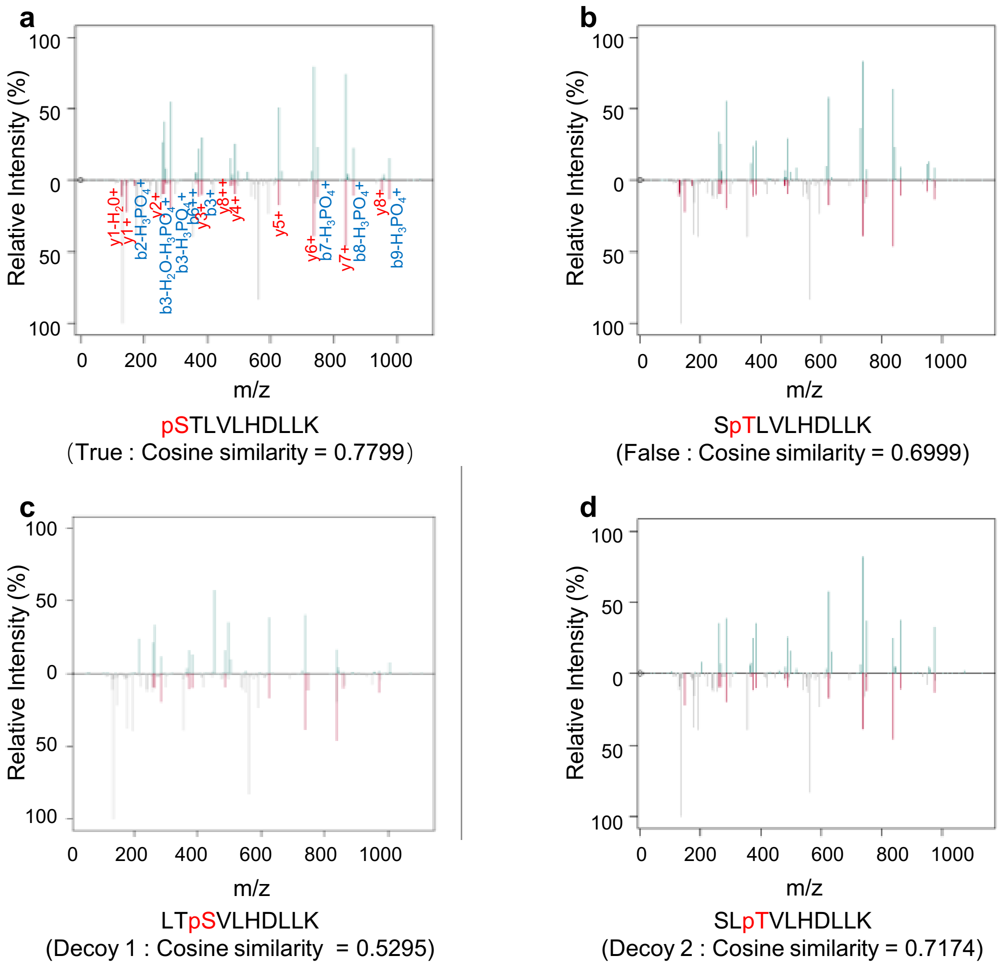

Supplementary Figure 14. Site localization of phosphopeptide pSTLVLHDLLK with charge state 2. Comparison of the experimental MS/MS with the predicted MS/MS of target and decoy phosphopeptides. Top: the predicted MS/MS spectra of **(a)** true target phosphopeptide pSTLVLHDLLK, **(b)** false target phosphopeptide SpTLVLHDLLK, **(c)** decoy phosphopeptide LTpSVLHDLLK and **(d)** decoy phosphopeptide SLpTVLHDLLK; Bottom: the experimental spectrum. Assignments in common are labeled in red color for the experimental spectrum.

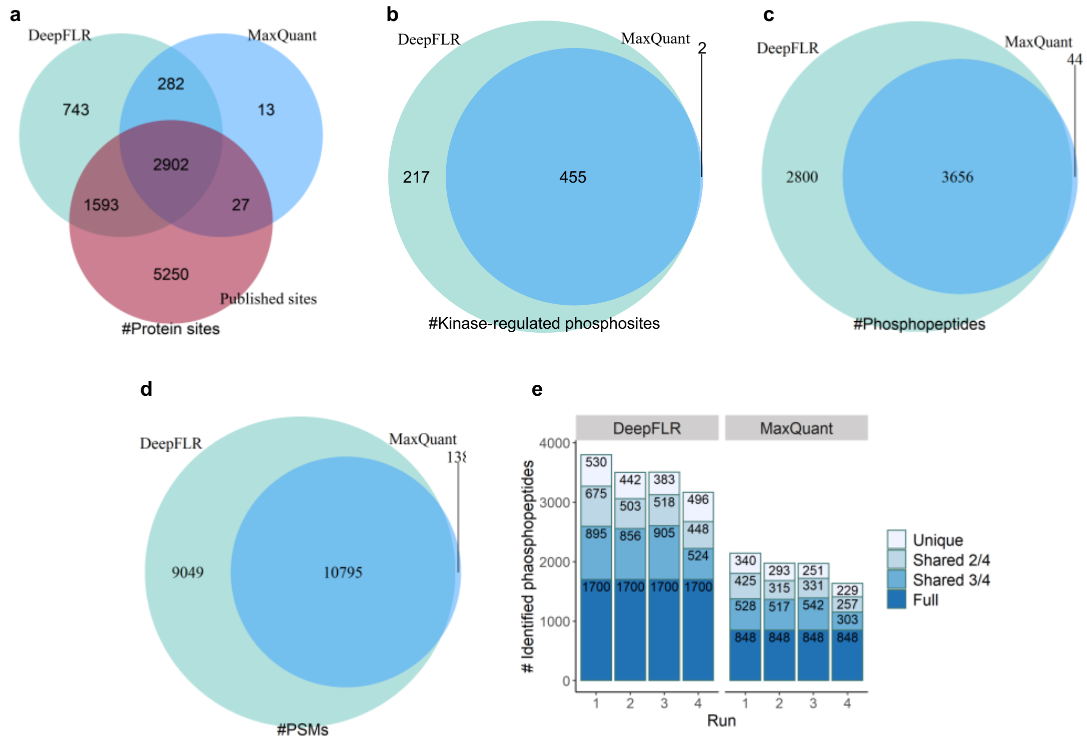

Supplementary Figure 15. Analysis of biological sample Bio_2 by DeepFLR (a) Venn diagram of the number of phosphosites localized by DeepFLR and MaxQuant based on the DDA data, as well as that reported by the original paper (Nat. Methods 13, 431-434 (2016)) combining the identification results of the sample by PRM, DDA and DIA. **(b)** Venn diagram of the number of kinase-regulated phosphosites identified by DeepFLR and MaxQuant. Venn diagram of the number of **(c)** phosphopeptides and **(d)** PSMs identified by DeepFLR and MaxQuant. **(e)** Numbers of cumulative identifications of phosphopeptides across runs. Full represents identifications observed in all the 4 runs; Shared 3/4 represents identifications observed in 3 runs; Shared 2/4 represents identifications observed in 2 runs; Unique represents identifications observed in only 1 run. DeepFLR reports identification with 1% estimated FLR, while MaxQuant reports identifications with 0.99 localization probability.

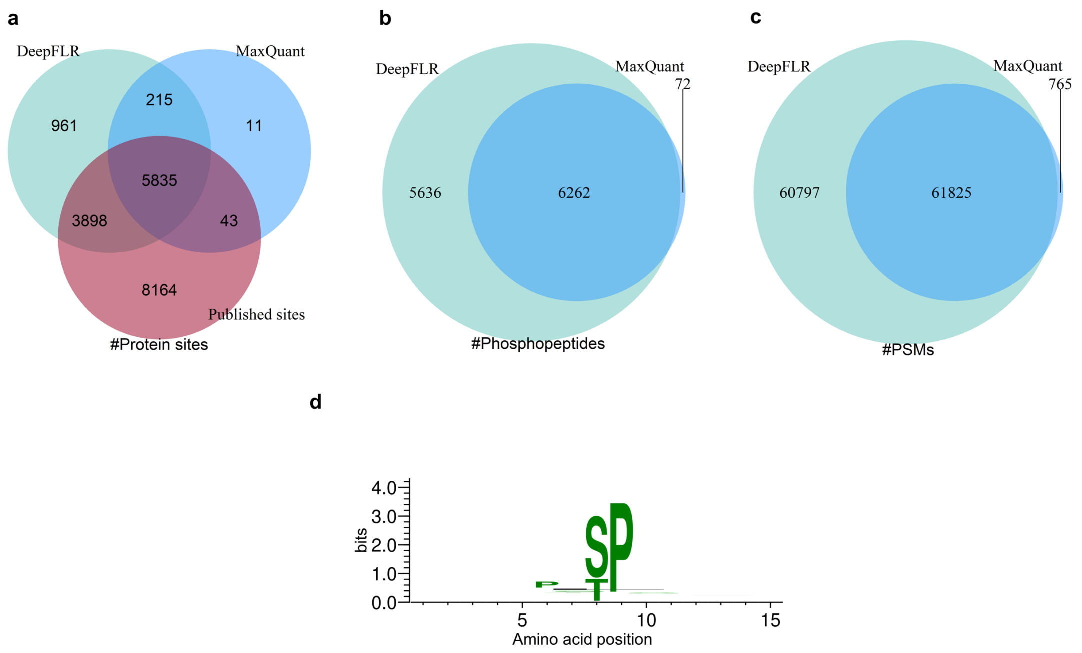

Supplementary Figure 16. Analysis of biological sample Bio_3 by DeepFLR. (a) Venn diagram of the number of phosphosites localized by DeepFLR and MaxQuant based on the DDA data, as well as that reported by the original paper (Nat. Commun. 11, 787 (2020).) combining the identification results of the sample by directDIA, DDA and DIA based on different spectral libraries. Venn diagram of the number of **(b)** phosphopeptides and **(c)** PSMs identified by DeepFLR and MaxQuant. **(d)** Amino acid sequence logo for the ERK 1/2 kinase substrate from PhosphoSitePlus (https://www.phosphosite.org/). Each column of the alignment is represented by a stack of letters where the height of each letter is proportional to the normalized frequency of the corresponding amino acid. DeepFLR reports identification with 1% estimated FLR, while MaxQuant reports identifications with 0.99 localization probability.

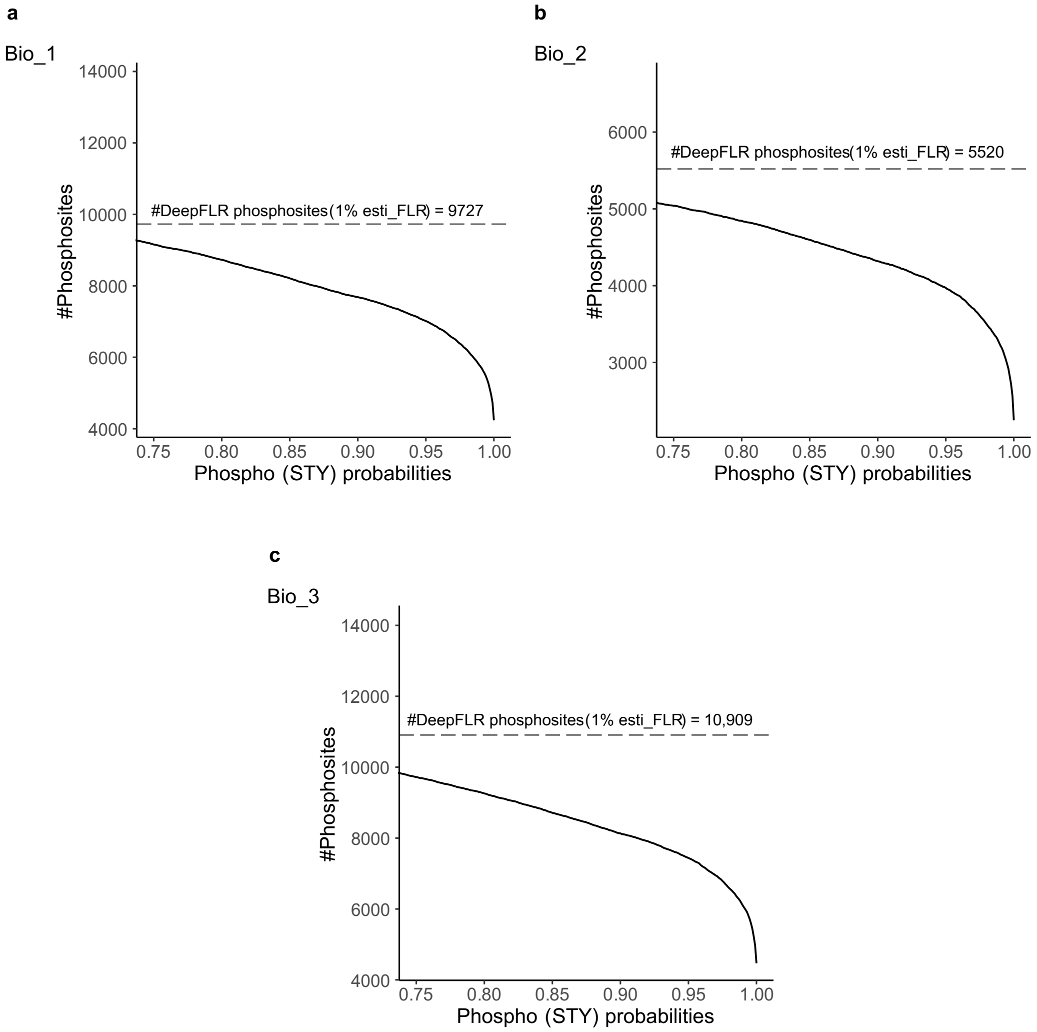

Supplementary Figure 17. Localization probability cutoff of MaxQuant plot against the number of localized protein phosphosites for **(a)** Bio_1, **(b)** Bio_2 and **(c)** Bio_3. The gray dashed horizontal line indicates the number of localized protein phosphosites at 1% estimated FLR by DeepFLR + MaxQuant for each dataset. Source data are provided as a Source Data file.

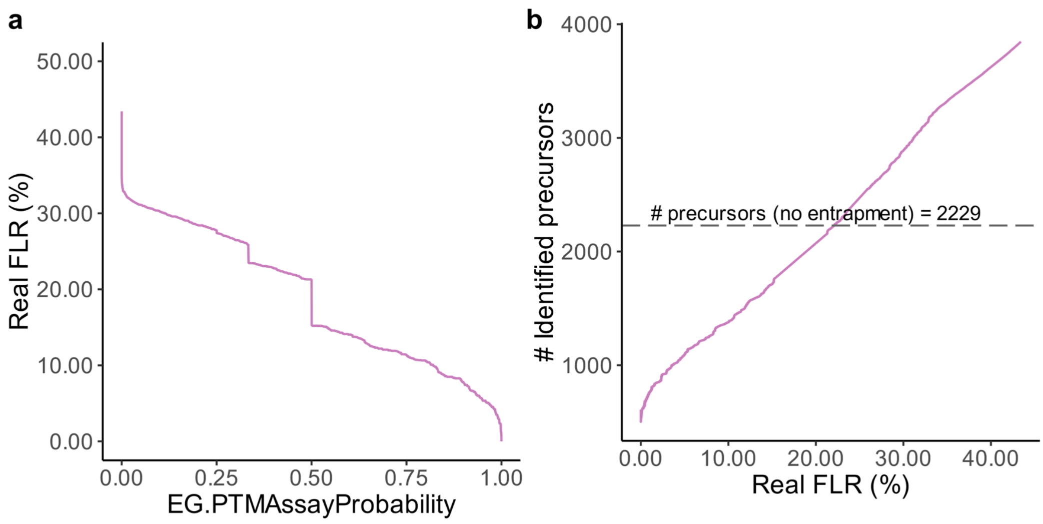

Supplementary Figure 18. (a) Correlation between real FLR and EG.PTMAssayProbability score by Spectronaut analysis of the synthetic phosphopeptides DIA dataset DIA_1, where the spectral library contains 1:1 target and entrapment phosphopeptides. The library contains all the synthetic phosphopeptides with correct phosphosites as the target phosphopeptides. Entrapment phosphopeptides are added to the library with correct peptide sequences but incorrect phosphosites. The precursor charge is 2 or 3. There are no additional PTMs besides Phosphorylation(STY). All the spectra of the target and entrapment phosphopeptides are predicted by DeepFLR and the retention time values are predicted by DeepPhospho. (b) Correlation between real FLR and identified phosphopeptides precursors when the library contains 1:1 target and entrapment phosphopeptides. The gray dashed horizontal line indicates the number of identified phosphopeptides precursors when the spectral library contains only target phosphopeptides. Source data are provided as a Source Data file.

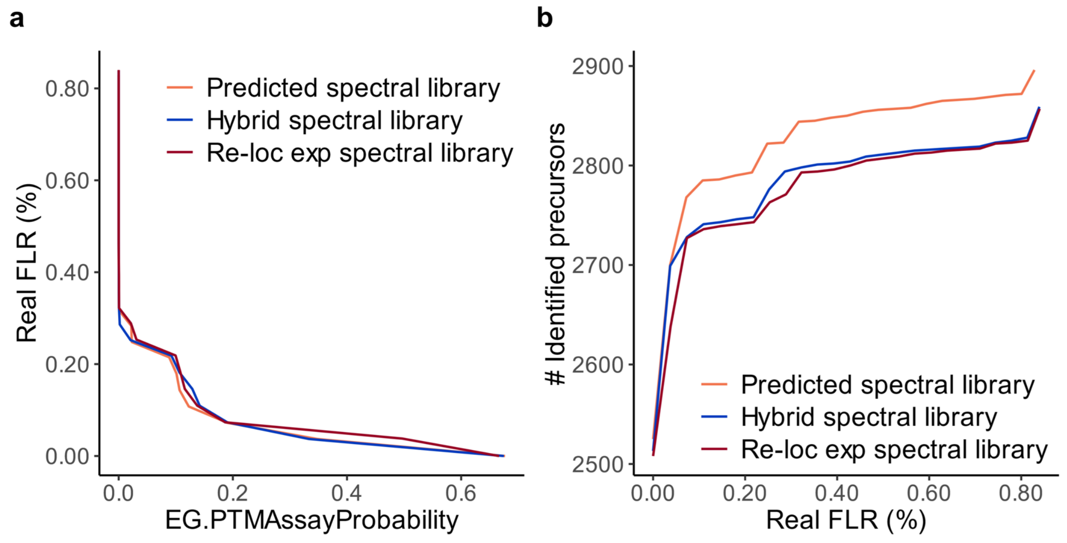

Supplementary Figure 19. (a) Correlation between real FLR and EG.PTMAssayProbability score by Spectronaut analysis of the synthetic phosphopeptides DIA dataset DIA_1 with the predicted spectral library, the hybrid spectral library and the re-localized experimental spectral library. The phosphopeptides in the libraries were obtained by DeepFLR analysis of the corresponding DDA data with 1% estimated FLR. (b) Correlation between real FLR and identified phosphopeptides precursors with the predicted spectral library, the hybrid spectral library and the re-localized experiment spectral library. Source data are provided as a Source Data file.

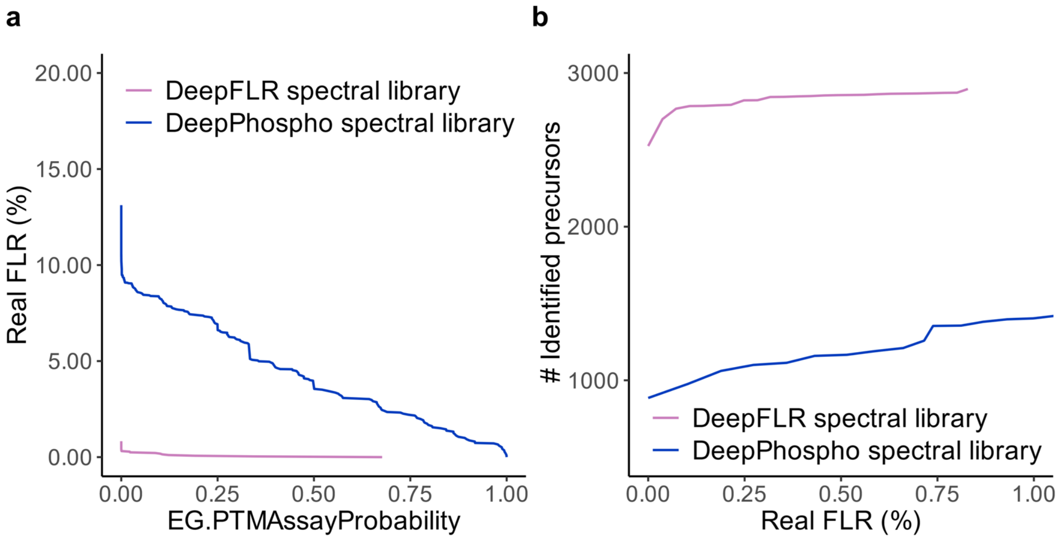

Supplementary Figure 20. (a) Correlation between real FLR and EG.PTMAssayProbability score by Spectronaut analysis of the synthetic phosphopeptides DIA dataset DIA_1 with the DeepFLR predicted spectral library and the DeepPhospho predicted spectral library. For the DeepFLR predicted spectral library, the phosphopeptides in the library were obtained by DeepFLR analysis of the corresponding DDA data with 1% estimated FLR. The MS/MS spectra were predicted by DeepFLR, and the retention time values were from experiments. For the DeepPhospho predicted spectral library, the phosphopeptides in the library were obtained by SpectroMine analysis of the corresponding DDA data with 0.75 localization probability threshold. Both the MS/MS spectra and the retention time values were predicted by DeepPhospho. (b) Correlation between real FLR and identified phosphopeptides precursors with the DeepFLR predicted spectral library and the DeepPhospho predicted spectral library. Source data are provided as a Source Data file.

Supplementary reference:

1. Bekker-Jensen, D. B. et al. An optimized shotgun strategy for the rapid generation of comprehensive human proteomes. *Cell Syst.* **4**, 587-599 (2017).
2. Kauko, O. et al. Label-free quantitative phosphoproteomics with novel pairwise abundance normalization reveals synergistic RAS and CIP2A signaling. *Sci. Rep.* **5**, 13099 (2015).
3. Kelstrup, C. D. et al. Rapid and deep proteomes by faster sequencing on a benchtop quadrupole ultra-high-field orbitrap mass spectrometer. *J. Proteome Res.* **13**, 6187-6195 (2014).
4. Haahr, P. et al. Activation of the ATR kinase by the RPA-binding protein ETAA1. *Nat. Cell Biol.* **18**, 1196-1207 (2016).
5. Wagner, S. A. et al. ATR inhibition rewires cellular signaling networks induced by replication stress. *Proteomics* **16**, 402-416 (2016).
6. Tsiatsiani, L. et al. Opposite electron-transfer dissociation and higher-energy collisional dissociation fragmentation characteristics of proteolytic K/R(X)(n) and (X)(n)K/R peptides provide benefits for peptide sequencing in proteomics and phosphoproteomics. *J. Proteome Res.* **16**, 852-861 (2017).
7. Sharma, K. et al. Ultradeep human phosphoproteome reveals a distinct regulatory nature of Tyr and Ser/Thr-based signaling. *Cell Rep.* **8**, 1583-1594 (2014).
8. van der Mijn, J. C. et al. Evaluation of different phospho-tyrosine antibodies for label-free phosphoproteomics. *J. Proteomics* **127**, 259-263 (2015).
9. Liu, Z. Y., Wang, F. J., Chen, J., Zhou, Y. & Zou, H. F. Modulating the selectivity of affinity absorbents to multi-phosphopeptides by a competitive substitution strategy. *J. Chromatogr. A* **1461**, 35-41 (2016).
10. Piersma, S. R. et al. Feasibility of label-free phosphoproteomics and application to base-line signaling of colorectal cancer cell lines. *J. Proteomics* **127**, 247-258 (2015).
11. Drake, J. M. et al. Phosphoproteome integration reveals patient-specific networks in prostate cancer. *Cell* **166**, 1041-1054 (2016).
12. Francavilla, C. et al. Phosphoproteomics of primary cells reveals druggable kinase signatures in ovarian cancer. *Cell Rep.* **18**, 3242-3256 (2017).
13. Nguyen, E. V. et al. Hyper-phosphorylation of sequestosome-1 distinguishes resistance to cisplatin in patient derived high grade serous ovarian cancer cells. *Mol. Cell. Proteomics* **16**, 1377-1392 (2017).
14. Ondrej, M., Rehulka, P., Rehulkova, H., Kupcik, R. & Tichy, A. Fractionation of enriched phosphopeptides using pH/acetonitrile-gradient-reversed-phase microcolumn separation in combination with LC-MS/MS analysis. *Int. J. Mol. Sci.* **21**, 3971 (2020).
15. Meul, T. et al. Mitochondrial Regulation of the 26S Proteasome. *Cell Rep* **32**, 108059 (2020).
16. Wu, X. N. et al. Sucrose-induced receptor kinase 1 is modulated by an interacting kinase with short extracellular domain. *Mol. Cell. Proteomics* **18**, 1556-1571 (2019).
17. Osman, S. et al. The Cdk8 kinase module regulates interaction of the mediator complex with RNA polymerase II. *J. Biol. Chem.* **296**, 100734 (2021).
18. Taumer, C. et al. Parallel reaction monitoring on a Q Exactive mass spectrometer increases reproducibility of phosphopeptide detection in bacterial phosphoproteomics measurements. *J. Proteomics* **189**, 60-66 (2018).
19. Ferries, S. et al. Evaluation of parameters for confident phosphorylation site localization using an Orbitrap Fusion Tribrid mass spectrometer. *J. Proteome Res.* **16**, 3448-3459 (2017).

20. Marx, H. et al. A large synthetic peptide and phosphopeptide reference library for mass spectrometry-based proteomics. *Nat. Biotechnol.* **31**, 557-564 (2013).
21. Bekker-Jensen, D. B. et al. Rapid and site-specific deep phosphoproteome profiling by data-independent acquisition without the need for spectral libraries. *Nat. Commun.* **11**, 787 (2020).
22. Shteynberg, D. D. et al. PTMProphet: fast and accurate mass modification localization for the Trans-Proteomic pipeline. *J. Proteome Res.* **18**, 4262-4272 (2019).
23. Rosenberger, G. et al. Inference and quantification of peptidoforms in large sample cohorts by SWATH-MS. *Nat. Biotechnol.* **35**, 781-788 (2017).
24. Takai, A. et al. Optimization of TripleTOF spectral simulation and library searching for confident localization of phosphorylation sites. *PLoS ONE* **14** 0225885 (2019).
25. Mehnert, M. et al. Multi-layered proteomic analyses decode compositional and functional effects of cancer mutations on kinase complexes. *Nat. Commun.* **11** 3563 (2020).
26. Lawrence, R. T., Searle, B. C., Llovet, A. & Villen, J. Plug-and-play analysis of the human phosphoproteome by targeted high-resolution mass spectrometry. *Nat. Methods* **13**, 431-434 (2016).
27. Searle, B. C., Lawrence, R. T., MacCoss, M. J. & Villen, J. Thesaurus: quantifying phosphopeptide positional isomers. *Nat. Methods* **16**, 703-706 (2019).
