# Supplementary material for: DeepFLR facilitates false localization rate control in phosphoproteomics
Source: Nat Commun. 2023 Apr 20;14:2269. doi: 10.1038/s41467-023-38035-1 (PMC10119288; doi:10.1038/s41467-023-38035-1)
Supplement: Supplementary file 2 — Description of Additional Supplementary Files [file 41467_2023_38035_MOESM2_ESM.docx]

File Name: Supplementary information

Description: Supplementary Tables and Figures.

File Name: Supplementary Data 1

Description: The protein phosphosites and phosphopeptides identified by DeepFLR and MaxQuant from the biological sample Bio_1.

File Name: Supplementary Data 2

Description: Phosphosites verified by targeted MS analysis.

File Name: Supplementary Data 3

Description: The protein phosphosites and phosphopeptides identified by DeepFLR and Maxquant from the biological sample Bio_2.

File Name: Supplementary Data 4

Description: The protein phosphosites and phosphopeptides identified by DeepFLR and Maxquant from the biological sample Bio_3.

File Name: Supplementary Data 5

Description: The significantly regulated sites identified from Bio_3 by DeepFLR and Maxquant.

File Name: Supplementary Data 6

Description: The protein phosphosites identified using DeepFLR-based predicted spectral library and SpectroMine-based experimental spectral library from DIA_2.

File Name: Supplementary Data 7

Description: Summary of the datasets used and the raw files used for each dataset from the public resources.
