# Supplementary material for: DeepFLR facilitates false localization rate control in phosphoproteomics
Source: Nat Commun. 2023 Apr 20;14:2269. doi: 10.1038/s41467-023-38035-1 (PMC10119288; doi:10.1038/s41467-023-38035-1)
Supplement: Supplementary file 10 — Reporting Summary [file 41467_2023_38035_MOESM10_ESM.pdf]

nature portfolio

Corresponding author(s): Liang Qiao

Last updated by author(s): Mar 28, 2023

# Reporting Summary

Nature Portfolio wishes to improve the reproducibility of the work that we publish. This form provides structure for consistency and transparency in reporting. For further information on Nature Portfolio policies, see our Editorial Policies and the Editorial Policy Checklist.

## Statistics

For all statistical analyses, confirm that the following items are present in the figure legend, table legend, main text, or Methods section.

| n/a | Confirmed | |
|---|---|---|
| ☐ | ☒ | The exact sample size (*n*) for each experimental group/condition, given as a discrete number and unit of measurement |
| ☐ | ☒ | A statement on whether measurements were taken from distinct samples or whether the same sample was measured repeatedly |
| ☐ | ☒ | The statistical test(s) used AND whether they are one- or two-sided<br>*Only common tests should be described solely by name; describe more complex techniques in the Methods section.* |
| ☒ | ☐ | A description of all covariates tested |
| ☒ | ☐ | A description of any assumptions or corrections, such as tests of normality and adjustment for multiple comparisons |
| ☐ | ☒ | A full description of the statistical parameters including central tendency (e.g. means) or other basic estimates (e.g. regression coefficient) AND variation (e.g. standard deviation) or associated estimates of uncertainty (e.g. confidence intervals) |
| ☐ | ☒ | For null hypothesis testing, the test statistic (e.g. *F*, *t*, *r*) with confidence intervals, effect sizes, degrees of freedom and *P* value noted<br>*Give P values as exact values whenever suitable.* |
| ☒ | ☐ | For Bayesian analysis, information on the choice of priors and Markov chain Monte Carlo settings |
| ☐ | ☒ | For hierarchical and complex designs, identification of the appropriate level for tests and full reporting of outcomes |
| ☐ | ☒ | Estimates of effect sizes (e.g. Cohen's *d*, Pearson's *r*), indicating how they were calculated |

*Our web collection on statistics for biologists contains articles on many of the points above.*

## Software and code

Policy information about availability of computer code

| | |
|---|---|
| Data collection | Data collection was performed by Thermo Xcalibur (version 3.0.63) and Bruker Compass HyStar 6.0. |
| Data analysis | The experimental spectra were converted to Mascot generic format (MGF) format using MsConvert from the ProteoWizard Package (3.0.11579). Raw data were analyzed by SpectroMine (version 2.5.201125, Biognosys AG, Schlieren, Switzerland), MaxQuant (version 1.6.17.0), PEAKS studio (version X+, Bioinformatics Solutions Inc., Waterloo, Canada), LuciPhOr2 (JAVA Version 8 Update 321), SpectroDive (version 11, Biognosys AG, Schlieren, Switzerland), Spectronaut Enterprise x6479 (version 16.1.220730.53000, Biognosys AG, Schlieren, Switzerland), AscorePro [https://github.com/gygilab/MPToolkit], phosphoRS (version 3.1), and our self built models. pDeep2 [https://github.com/pFindStudio/pDeep/tree/master/pDeep2] and DeepPhospho [https://github.com/weizhenFrank/DeepPhospho] were used for phosphopeptides MS/MS spectra prediction to compare with DeepFLR. Model construction was performed using python (3.8.3) with the following packages: FastNLP (0.6.0), pytorch (1.8.1), bidict (0.22.0), pyteomics (4.5.5) and transformers (4.12.5). Data analysis for FLR estimation was performed using python (3.8.3) with the following packages: pandas (1.0.5) and numpy (1.18.5). For biological samples, the sequence logo was constructed by WebLogo (3.7.4) and the biological sample quantitative analysis was performed with Perseus (1.6.15.0). Visualization was performed using custom scripts in R (4.0.2) with the following packages: VennDiagram (1.6.20), ComplexHeatmap (2.13.2) and ggplot2 (3.3.2). DeepFLR is open source and freely available on GitHub [https://github.com/lmsac/DeepFLR] and Zenodo [https://doi.org/10.5281/zenodo.7777409]. |

For manuscripts utilizing custom algorithms or software that are central to the research but not yet described in published literature, software must be made available to editors and reviewers. We strongly encourage code deposition in a community repository (e.g. GitHub). See the Nature Portfolio guidelines for submitting code & software for further information.

## Data

Policy information about availability of data

All manuscripts must include a data availability statement. This statement should provide the following information, where applicable:

- Accession codes, unique identifiers, or web links for publicly available datasets
- A description of any restrictions on data availability
- For clinical datasets or third party data, please ensure that the statement adheres to our policy

All the LC-MS/MS raw data, FASTA files, search results, saved projects and database searching parameters generated in this study have been deposited to ProteomeXchange via the iProX partner repository under accession code PXD037580 [http://proteomecentral.proteomexchange.org/cgi/GetDataset?ID=PXD037580] or IPX0005248000 [https://www.iprox.cn/page/project.html?id=IPX0005248000]. The 14 raw datasets used in this study for deep learning model pre-training are available in the PRIDE database under accession code PXD004452 [http://proteomecentral.proteomexchange.org/cgi/GetDataset?ID=PXD004452], PXD001374 [http://proteomecentral.proteomexchange.org/cgi/GetDataset?ID=PXD001374], PXD001305 [http://proteomecentral.proteomexchange.org/cgi/GetDataset?ID=PXD001305], PXD003529 [http://proteomecentral.proteomexchange.org/cgi/GetDataset?ID=PXD003529], PXD002135 [http://proteomecentral.proteomexchange.org/cgi/GetDataset?ID=PXD002135], PXD004447 [http://proteomecentral.proteomexchange.org/cgi/GetDataset?ID=PXD004447], PXD000612 [http://proteomecentral.proteomexchange.org/cgi/GetDataset?ID=PXD000612], PXD001565 [http://proteomecentral.proteomexchange.org/cgi/GetDataset?ID=PXD001565], PXD004252 [http://proteomecentral.proteomexchange.org/cgi/GetDataset?ID=PXD004252], PXD001550 [http://proteomecentral.proteomexchange.org/cgi/GetDataset?ID=PXD001550], PXD001546 [http://proteomecentral.proteomexchange.org/cgi/GetDataset?ID=PXD001546], PXD002286 [http://proteomecentral.proteomexchange.org/cgi/GetDataset?ID=PXD002286], PXD003531 [http://proteomecentral.proteomexchange.org/cgi/GetDataset?ID=PXD003531] and PXD002394 [http://proteomecentral.proteomexchange.org/cgi/GetDataset?ID=PXD002394]. The three datasets used in this study for model re-training to fit the Q-TOF data are available in the PRIDE database under accession code PXD006056 [http://proteomecentral.proteomexchange.org/cgi/GetDataset?ID=PXD006056], PXD012433 [http://proteomecentral.proteomexchange.org/cgi/GetDataset?ID=PXD012433] and PXD015687 [http://proteomecentral.proteomexchange.org/cgi/GetDataset?ID=PXD015687]. The five datasets used in this study for evaluating the performance of DeepFLR in MS/MS spectra prediction are available in the PRIDE database with the accession number PXD018663 [http://proteomecentral.proteomexchange.org/cgi/GetDataset?ID=PXD018663] (Test_1), PXD019697 [http://proteomecentral.proteomexchange.org/cgi/GetDataset?ID=PXD019697] (Test_2), PXD011284 [http://proteomecentral.proteomexchange.org/cgi/GetDataset?ID=PXD011284] (Test_3), PXD023361 [http://proteomecentral.proteomexchange.org/cgi/GetDataset?ID=PXD023361] (Test_4) and PXD008211 [http://proteomecentral.proteomexchange.org/cgi/GetDataset?ID=PXD008211] (Test_5). The four synthetic phosphopeptides datasets used in this study to evaluate the performance of DeepFLR in FLR control are available in the PRIDE database with the accession number PXD007058 [http://proteomecentral.proteomexchange.org/cgi/GetDataset?ID=PXD007058] (Syn_1), PXD000138 [http://proteomecentral.proteomexchange.org/cgi/GetDataset?ID=PXD000138] (Syn_2), PXD014525 [http://proteomecentral.proteomexchange.org/cgi/GetDataset?ID=PXD014525] (Syn_3) and PXD013210 [http://proteomecentral.proteomexchange.org/cgi/GetDataset?ID=PXD013210] (Syn_4). Two external biological datasets used in this study to evaluate the performance of DeepFLR in the analysis of biological samples are available in the PRIDE database with the accession number PXD003344 [http://proteomecentral.proteomexchange.org/cgi/GetDataset?ID=PXD003344] (Bio_2) and PXD014525 [http://proteomecentral.proteomexchange.org/cgi/GetDataset?ID=PXD014525] (Bio_3). Two DIA datasets used in this study to evaluate the performance of DeepFLR in DIA analysis are available in the PRIDE database with the accession number PXD014525 [http://proteomecentral.proteomexchange.org/cgi/GetDataset?ID=PXD014525] (DIA_1) and from MassIVE proteomics repository with project identifier MSV000082956 [ftp://massive.ucsd.edu/MSV000082956/] (DIA_2). The source data underlying all figures except for those not including statistics are provided as a Source Data file.

## Human research participants

Policy information about studies involving human research participants and Sex and Gender in Research.

| | |
|---|---|
| Reporting on sex and gender | Not applicable. This study doesn't involve human research participants. |
| Population characteristics | Not applicable. This study doesn't involve human research participants. |
| Recruitment | Not applicable. This study doesn't involve human research participants |
| Ethics oversight | Not applicable. This study doesn't involve human research participants |

Note that full information on the approval of the study protocol must also be provided in the manuscript.

# Field-specific reporting

Please select the one below that is the best fit for your research. If you are not sure, read the appropriate sections before making your selection.

☒ Life sciences ☐ Behavioural & social sciences ☐ Ecological, evolutionary & environmental sciences

For a reference copy of the document with all sections, see nature.com/documents/nr-reporting-summary-flat.pdf

# Life sciences study design

All studies must disclose on these points even when the disclosure is negative.

| | |
|---|---|
| Sample size | This study doesn't involve any biological discovery. Phosphopeptides enriched from the tryptic digests of proteins from Hela cells were |

| | |
|---|---|
| | analyzed by LC-MS/MS with 3 technical replicates. This sample size is usually used in proteomics study to validate technical variance. |
| Data exclusions | No data were excluded. |
| Replication | Phosphopeptides enriched from the tryptic digests of proteins from Hela cells were analyzed by LC-MS/MS with 3 technical replicates to test the identification and quantification performance of DeepFLR. All attempts at replication were successful. |
| Randomization | Not applicable. This study doesn't involve any biological discovery and the performance of phosphopeptides identification/quantification by DeepFLR was tested on all the datasets. No randomization was carried out. |
| Blinding | Not applicable. This study focuses on technical strength of our proposed method and does not report any biological findings. The samples used in this study are ordinary model specimens without labeling, grouping or classification. Therefore, no blinding was carried out. |

# Reporting for specific materials, systems and methods

We require information from authors about some types of materials, experimental systems and methods used in many studies. Here, indicate whether each material, system or method listed is relevant to your study. If you are not sure if a list item applies to your research, read the appropriate section before selecting a response.

## Materials & experimental systems

| n/a | Involved in the study | |
|---|---|---|
| ☒ | ☐ | Antibodies |
| ☐ | ☒ | Eukaryotic cell lines |
| ☒ | ☐ | Palaeontology and archaeology |
| ☒ | ☐ | Animals and other organisms |
| ☒ | ☐ | Clinical data |
| ☒ | ☐ | Dual use research of concern |

## Methods

| n/a | Involved in the study | |
|---|---|---|
| ☒ | ☐ | ChIP-seq |
| ☒ | ☐ | Flow cytometry |
| ☒ | ☐ | MRI-based neuroimaging |

# Eukaryotic cell lines

Policy information about cell lines and Sex and Gender in Research

| | |
|---|---|
| Cell line source(s) | Human epithelial cervical cancer HeLa cells (HeLa, CCL-2) were obtained from the American Type Culture Collection (ATCC). |
| Authentication | This cell line was obtained within 6 months before being used in this study. The study doesn't involve biological discovery, and the cells were only used to test the ability of DeepFLR in phosphopeptides identification. The HeLa cells were not further authenticated after obtained from ATCC. |
| Mycoplasma contamination | We confirm that the cells used in this study are not contaminated by mycoplasma. |
| Commonly misidentified lines (See ICLAC register) | No commonly misidentified cell lines are used in this study. |
